# *Caenorhabditis elegans* Bacterial Pathogen Resistant *bus-4* Mutants Produce Altered Mucins

**DOI:** 10.1371/journal.pone.0107250

**Published:** 2014-10-08

**Authors:** Lisa M. Parsons, Rahman M. Mizanur, Ewa Jankowska, Jonathan Hodgkin, Delia O′Rourke, Dave Stroud, Salil Ghosh, John F. Cipollo

**Affiliations:** 1 Food and Drug Administration, Center for Biologics Evaluation and Research, Bethesda, Maryland, United States of America; 2 Genetics Unit, Department of Biochemistry, University of Oxford, Oxford, United Kingdom; Swiss Institute of Bioinformatics, Switzerland

## Abstract

*Caenorabditis elegans bus-4* glycosyltransferase mutants are resistant to infection by *Microbacterium nematophilum*, *Yersinia pestis* and *Yersinia pseudotuberculosis* and have altered susceptibility to two *Leucobacter* species Verde1 and Verde2. Our objective in this study was to define the glycosylation changes leading to this phenotype to better understand how these changes lead to pathogen resistance. We performed MALDI-TOF MS, tandem MS and GC/MS experiments to reveal fine structural detail for the *bus-4 N*- and *O*-glycan pools. We observed dramatic changes in *O*-glycans and moderate ones in *N*-glycan pools compared to the parent strain. *Ce* core-I glycans, the nematode's mucin glycan equivalent, were doubled in abundance, halved in charge and bore shifts in terminal substitutions. The fucosyl *O*-glycans, *Ce* core-II and neutral fucosyl forms, were also increased in abundance as were fucosyl *N*-glycans. Quantitative expression analysis revealed that two mucins, *let-653* and *osm-8*, were upregulated nearly 40 fold and also revealed was a dramatic increase in GDP-Man 4,6 dehydratease expression. We performed detailed lectin binding studies that showed changes in glycoconjugates in the surface coat, cuticle surface and intestine. The combined changes in cell surface glycoconjugate distribution, increased abundance and altered properties of mucin provide an environment where likely the above pathogens are not exposed to normal glycoconjugate dependent cues leading to barriers to these bacterial infections.

## Introduction

*Caenorhabditis elegans* can be infected by over forty microbial pathogens [1]. Among these are the nematode specific pathogen, *Microbacterium nematophilum*, and the human pathogens *Yersinia pestis* and *Yersinia pseudotuberculosis*. *M. nematophilum* infects the anus, rectum and surrounding cuticle of the nematode causing localized swelling and constipation [2]. *Y. pestis* and *Y. psudotuberculosis* do not directly infect *C. elegans*. Rather these bacteria secrete an exopolysaccharide that adheres to the head region of the nematode, causing starvation [3]. The *bus* and *bah* genetic screens have isolated mutants resistant to *M. nematophilum* and *Yersinia* spp. respectively, and there is significant genetic overlap between the screens demonstrating that a series of the same genes are required for both pathogenic processes.

The *bus* screens have yielded more than 20 complementation groups [4], [5]. These were characterized by an absence of swelling in the tail region when exposed to the *M. nematophilum* leading to the bacterially *u*n*s*wollen (*bus*) phenotype. Included among the genes that have been cloned six (*bus-2*, *bus-4*, *bus-8*, *bus-12*, *bus-17* and *srf-3*) encode a distinct gene required for, glycoconjugate biosynthesis [6]-[8]. The *bus-2*, *bus-4* and *bus-17* genes encode homologs of glycosyltransferases [8]. The *bus-12* [8] and *srf-3* [9] genes encode nucleotide sugar transporter homologs and *bus-8* encodes a mannosyltransferase homolog predicted to act in protein *N*-glycosylation [6]. A screen for altered susceptibility to *Yersinia* biofilm attachment to the head region (biofilm absent on head, *bah*) identified eight genes. Five genes identified in these *bah* screens were also identified in *bus* screens. These were *srf-2*, *srf-3*, *bus-4*, *bus-12* and *bus-17* [7]. The *srf-2* and *srf-3* mutants were originally selected in screens by their ectopic lectin binding *srf* (surface) [10], [11] phenotype. That screening with bacterial pathogens and lectins identified the same genes infers similarities in the mutant phenotypes, namely a shift in glycoconjugate status at the cuticle surface. All of these screens identified genes required for glycosylation of the cuticle and these genes are expressed in the hypodermal seam cells demonstrating that these cells are required for surface coat and/or cuticle glycoconjugate production [5], [8].

The genetic overlap between the *bus*, *bah* and *srf* screens directs attention to the importance of glycoconjugates in the *C. elegans* cuticle. Glycosylation defects imposed by these defects disrupt cuticle surface interactions with pathogens and other interactions as well. *Bacillus pumilus* secretes an adhesive that can trap wild type *C. elegans* [8]. In *B. pumilus* hurdle assays *bus-4*, *bus-8*, *bus-17* and *srf-3* pass over the adhesive zone without difficulty, while *bus-2* and *bus-12* have increased difficulty. In all of these mutants, male interaction with wild type hermaphrodites is defective. The *bus-4*, *bus-8*, *bus-12* and *bus-17* have a skiddy movement on *E. coli* lawns and all but *bus-12* are fragile [7]. Additionally, the *bus-4* mutants demonstrate altered susceptibilities to the *Leucobacter* isolates, referred to as Verde 1 and Verde 2 [12]. While Verde-1 is non-lethal to wildtype nematodes, it is lethal to *bus*-4 nematodes. Alternatively, Verde-2 is lethal to wildtype worms but non-lethal to *bus*-4 nematodes. All of these characteristics demonstrate that the cuticle of these mutants is altered through compromised glycosylation affecting how they interact with their environment.

Previously we reported the *N*- and *O*-glycan structures of the *srf-3* and *bus-2* reference strains [13], [14]. In *srf-3* mutants we observed a dramatic decrease in *Ce* core-I *O*-glycans and a marked loss of galactosyl *N*-glycans consistent with the function of *SRF-3*, a Golgi UDP-Gal/UDP-GalNAc multi- nucleotide sugar transporter [14]. In *bus-2* mutants, deficient in a predicted galactosyltransferase, we also found changes in *O*-glycosylation but no significant changes in *N*-glycosylation. The *Ce* core-II *O*-glycans were revealed in that study as they were more abundant in the *bus-2* genetic background than wild-type or other strains previously examined. *Ce* core I glycans were also affected by the *bus-2* deficiency. Lectin studies of acetone fixed *bus-2* nematodes revealed a dramatic loss in *Ce* core-I *O*-glycoproteins over the cuticle surface as detected by ABA lectin staining. This glycoconjugate loss was especially dramatic in the anal and rectal regions and surrounding cuticle, the areas infected by *M. nematophilum*. Mass spectrometry analysis showed that the *Ce* core-I glycan pool compositions were also altered in distribution. Additionally, UEA-1 alpha fucose detecting lectin stained strongly in the distal intestine of *bus-2* nematodes. This signal was not susceptible to PNGase F treatment suggesting an aberrant or increased presence of fucosyl *O*-glycans in the hind gut, likely the *Ce* core-II *O*-glycoproteins. The evidence derived from the *srf-3* and *bus-2* studies strongly suggested that the infective processes of *M. nematophilum*, *Y. pestis* and *Y. pseudotuberculosis* requires proper *O*-glycosylation.

In the current study we investigate the glycosylation defect in the *bus-4* reference strain. We have performed lectin binding studies of both fixed and live nematodes and detailed structural analyses of released *N*- and *O*-linked glycans. We also performed quantitative expression analyses of key pathway glycosylation enzymes. These studies reveal a unique *bus-4* glycomics profile at the levels of glycan fine structure, tissue specific distributions and related expression levels. These changes impact interaction between *C. elegans* and its environment including the pathogenic interactions with *M. nematophilum*, *Y. pestis* and *Y. pseudotuberculosis*. The impact these changes have on these interactions is discussed.

## Materials and Methods

### Strains and culture maintenance

*C. elegans* wild type (N2 Bristol), which is sensitive to *M. nematophilum* infection, was used as control. The *bus-4* strain (*e2693*) used in this study was generated by Ethylmethane Sulphonate mutagenesis [4]. It contains a missense mutation, Gly224Arg, which changes a highly conserved residue in the galactosyltransferase domain, and is therefore likely to be null or near-null for enzyme activity. A different allele, *e2700*, was used in some rescue and lectin staining experiments; this allele is phenotypically similar to *e2693* and changes a residue (Gly58Asp) conserved between different *Caenorhabditis* species. Both of the N2 and *bus-4* strains were provided by JH. *C. elegans* strains were maintained at 16°C on NGM agar seeded with *Escherichia coli* OP50 according to the standard methods as described previously [15].

### Bacterial strains and growth conditions

*E. coli* OP50 and *M. nematophilum* strains CBX102 were grown in Luria Bertani (LB) nutrient medium at 37 °C for 24 h and 48 h, respectively. For assessment of *C. elegans* susceptibility to infection Nematode Growth Medium (NGM) agar plates were aseptically seeded with the *E. coli* strain OP50 containing 1% *M. nematophilum* CBX102 as described previously [4].

### Transgenic rescue of bus-4

Construction of strain CB6817 is described in more detail elsewhere [16]. This strain is homozygous for a *bus-4* mutation, allele *e2700*, and carries a transgene containing an operon fusion of wildtype *bus-4* with *mCherry*, together with *sur-5::GFP* as a transformation marker.

### Quantitative RT-PCR (qRT-PCR) analysis

Transcript levels of *gmd-2*, *osm-8*, *let-653*, *lipl-1*, *bus-2*, *gly-11*and *bus-8* transgenes in N2 and *bus-4* strains, were measured by quantitative RT-PCR. The following primers were used: gmd-2 For AAAGCGAGCTGACCCCATT; gmd-2 rev ATACATCTTGGCGACCGCATA; let 653 For CTGTCTCGTGAGAATATGTCC; let-653-Rev TTCCACGTCGTCGCATGT; osm-8 For AGAAGCCCCACCACTGATTG; osm-8 Rev TTGTTTTTGCCACGGTTCAA, lipl-1 For GCGGATCCGGAGATGAAGA

lipl-1 Rev GGATATCCCCATCGCATGAT, bus-2 For AGTTTCCCGACGGATTCCA, bus-2 Rev AGGGCGATGAGGCAACTG, gly-11 For GGACCTGCGGTGGAGAACT, gly-11 Rev GCGGAAAATGTGGCCAACT, bus-8 For CTCCGGACCAGAAGCTAGGA, bus-8 Rev CGCCCTCCAATGAAAGTCATTotal RNA was obtained from the biological triplicate of each strain. The cDNA was produced from the total RNA extracts using a random primer strategy and Superscript III reverse transcriptase. Primers were designed by the program primer 3, where amplicon size was restricted to 60 mer. Primers were ordered from IDT. Finally expression of the transgenes were determined quantitatively by the relativeCt method using SYBR green (from Applied Biosystem) as fluorescent detector in an applied biosystem 7900 HT instrument Results are the average of three biological replicates normalized to the geometric mean of one control gene (*actin 4*). Changes were determined by normalizing samples to a control sample (*N2*). Error bars represent one standard deviation.

### Large scale C. elegans culture

The *C. elegans* strains were grown in 2.8 liter Fernbach Flasks containing 250 ml of M9 media (3 g/l KH_2_PO_4_, 6 g/l Na_2_HPO_4_, 5 g/l NaCl, 1 mM MgSO_4_), 5 mg/L cholesterol, and 7 grams of *E. coli* OP50 to serve as a food source. Worms washed from three NGM plates (100 mm x 15 mm) were used to inoculate each culture which was gently shaken at 75 rpm at room temperature for 3-4 days until the worms were visible when the flask was tipped.

Nematode cultures were harvested by placing the flasks on ice in a slightly tipped position about 30 minutes until the worms had settled to the edge. Then the media was poured off. The remaining pellet was split into two 50 ml Falcon tubes and washed three times with cold 100 mM NaCl by centrifugation at 400 x g for 2 – 3 minutes in a spinning bucket centrifuge. The worms were rocked in 100 mM NaCl at room temperature for 30 minutes and pelleted to remove debris. The drops of worms were then flash frozen with liquid nitrogen and the pellets stored at 80°C. Cultures were processed in this manner until 10 to 12 grams (wet weight) of nematodes for each strain were collected.

### Glycoprotein preparation

Flash frozen nematode pellets were processed for isolation of glycoprotein rich extracts as previously described [13].

### Glycan release and derivatization

*C. elegans* glycoprotein enriched extracts were processed for sequential release of *N*- and *O*-glycans using PNGase F and PNGase A for the former and β-elimination for the latter as previously described [13]. PNGase F was used first to release the majority of N-glycans while PNGase A was used subsequently to release retained *N*-glycans which are enriched for fucosyl forms in *C. elegans* preparations. All released glycans were permethylated for structural analysis as previously described by Ciucanu *et al*. [17], [18].

### MALDI-TOF MS Analysis

Glycans were permethylated according to Ciucanu *et al*., The permethylated glycans [18] were suspended in 50% methanol/water, spotted onto a MALDI plate, mixed 1∶1 with 2,5-DHB matrix in the same solution plus 1 mM sodium acetate, and analyzed using a Perseptive Biosystems Voyager DE RF MALDI-TOF mass spectrometer. Samples were analyzed in positive ion reflectron mode in the 800-5500 m/z range in triplicate. Matched N2 and *bus-4* glycan samples were analyzed. Three hundred individual scans were summed per analysis. The MS data were processed with DataExplorer (Perseptive Biosystems).

### Reductive Amination

Oligosaccharides were dried in a Savant speed evacuation device and reconstituted in 15 µl of dimethylsulfoxide (DMSO). To the reconstituted samples was added 100µl of 2 M cyanoborohydride and 35 µl of 0.5 M 2-amino benzamide (2-AB), both of which were solubilized in DMSO. Glacial acetic acid (50 µl) was then added. The reaction was performed at 65°C for 2 h. The reaction mixture was then diluted to one ml with 90% acetonitrile containing 0.1% trifluoroacetic acid (TFA) and was applied to a Amide-80 (HILIC, Tosoh, Japan) column that was preequilibrated with 90% acetonitrile containing 0.1% TFA. The columns were then washed three times with one ml 90% acetonitrile containing 0.1% TFA and finally the aminated glycans were eluted with one ml of 50% acetonitrile containing 0.1% TFA, dried and stored at -20 °C.

### Ion Trap LC/MS Analysis of Aminated Oligosaccharides

The characterization of *N*-glycans released from *C. elegans* and labeled with 2-AB was carried out by LC/MS analysis on an LTQ XL linear ion trap mass spectrometer equipped with an ESI source (Thermo-Electron, San Jose, CA, USA). The mass spectrometry coupled liquid chromatography was performed using a Surveyor autosampler plus and MS pump plus (Thermo Scientific). The column used was a 1.0 mm x 15 cm, 5 µm TSK gel Amide-80 column (Tosoh Bioscience LLC, PA, USA). Solvent A was 20 mM ammonium acetate (pH 6.9) and solvent B was 100% acetonitrile. The flow rate was 40 µl/min. Initial conditions were 20% solvent A/80% solvent B. After 5 minutes solvent B was decreased from 80% to 25% over 100 minutes followed by re-equilibration for 10 minutes at initial conditions.

Mass spectrometer conditions were a spray voltage of 5.0 kV and the capillary temperature was 170°C. The sheath gas flow was set to 20.00 units. For the generation of the MS^n^ spectra, normalized collision energies were set to 35%. The method used was triple play operated by Xcalibur software, with second scan being Zoom MS and third scan was Dependent MS/MS of most intense ion from scan event 2. The isolation width was set to 2 amu. All experiments were performed in the positive ion mode.

### Ion Trap MS Analysis of Permethylated Oligosaccharides

Samples were dissolved in 40% methanol/10 % isopropanol/0.1 % FA. The Advion Nanomate (Ithaca, NY USA) source was used in direct infusion mode. The nitrogen gas pressure was set to 0.3 psi and spray voltage at 1.4 kV. Capillary temperature was maintained at 175 °C. MS^n^ spectra were collected at 50 scans each and normalized collision energy was set to 30%. Analyses were performed in triplicate.

### Calculation of substructure abundances

The *bus-4* and N2 glycans were analyzed under identical conditions allowing for direct comparison of spectral attributes. Based on relative abundances of compositions from MALDI-TOF MS and of unique daughter ion abundances from ion trap analyses produced from isomers within each composition an apparent abundance of each of the following substructures was calculated. Shown in parenthesis are the unique daughter ions used in the calculations: Glcβ1-4,6 (*m/z* 463.3), Glc/Galβ1-6GalNAc (*m/z* 502.3), Glcβ1,4GalNAc (*m/z* 706.5); GlcA1,4GalNAc (*m/z* 720.5); Glcβ1,3Gal (*m/z* 681.4 + *m/z* 885.5). Analyses were performed in triplicate and overall standard deviations calculated. Results are reported in mol/mol of core-I saccharide.

### GC/MS

*O*-glycans were aliquotted and dried with an internal standard of myoinositol in 2 ml heat resistant screw cap glass vials. Samples were prepared following the method of Kamerling *et al*.,(1975) [19]. In brief, 400 µl 1N Methanolic-HCL (diluted from Supelco cat. no. 3-3051 with anhydrous methanol) was added to each sample under nitrogen and tightly sealed. The samples were incubated in a shaking (350 rpm) heat block for 24 hours at 80°C. Solid silver carbonate was added until the samples were neutralized as monitored by pH paper. Acetic anhydride (40 µl) was then added and the samples were incubated at room temperature in the dark overnight. The next day, the samples were thoroughly mixed and the precipitates pelleted by centrifugation. The supernatants were saved and the precipitates were washed twice with 500 µl anhydrous methanol. The supernatant and the washes from each sample were pooled and dried by rotary evaporation. Tri-Sil HTP reagent (Thermo cat. no. TS-48999) (25 µl) was added to the dried samples under nitrogen and they were incubated at room temperature for thirty minutes. Finally, 50 µl hexane and 5 µl of dry hexane-washed Sephadex (as prepared by Elwood et al. 1988) [20] were added. The samples were vortexed, centrifuged and the Sephadex pellet removed. Approximately 40-50 µl of liquid was transferred to the GC/MS sample vial. Standard mixtures of four to six carefully weighed control monosaccharides were prepared in the same manner and included the same internal standard.

Samples (2 µl) were injected into a 30 m x 0.25 mm x 0. 25 µm Agilent J&W column (cat. no. 19091S-433) in an Agilent GC/MS (Models 7890A and 5975C) equipped with a GC Sampler 80. The inlet temperature was 300°C and the oven temperature was 80°C. After a 2 minute pause, the oven temperature ramped to 180°C at a rate of 20°C/min and was held at 180°C for 23 minutes. The carbohydrate abundances in each sample were calculated as described by Elwood *et al*. [20].

### Lectin staining of acetone fixed nematodes

**Figure 1:**
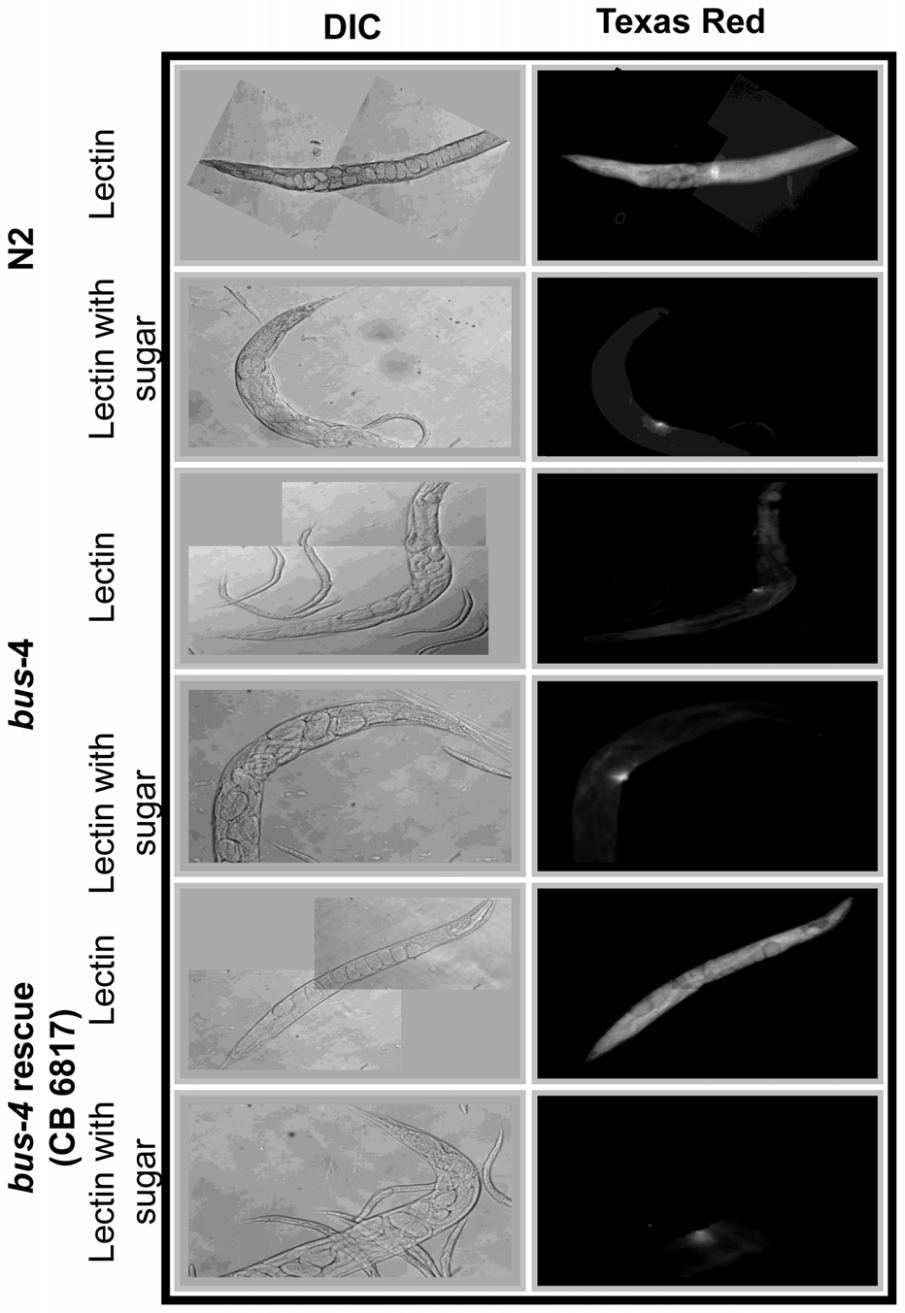
Lectin staining patterns of fixed *bus-4* nematodes are altered. Texas Red-conjugated *Agaricus bisporus* (ABA) Gal (β1,3) GalNAc specific lectin staining of whole mounted delipidated and live *C. elegans* strains are shown. A: the left columns shows differential interference contrast (DIC) micrographs and right columns show fluorescence micrographs of ABA stained fixed nematodes with and without prior incubation with inhibitory sugar (β-D-galactose). Top panels are N2 Bristol. Bottom panels are *bus-4*. B; Shorter exposures of ABA lectin are shown along with DIC micrographs to the left. Cuticle staining seen in the ventral tail region leading up to the anus in the N2 parent is absent in the *bus-4* strain. C; Live ABA stained nematodes are shown. The surface coat of the *bus-4* nematodes stains more intensely than in the N2 parent. The staining is most concentrated in the head and tail regions. Staining in the intestine is also increased abundances of soluble mucin-like proteins are indicated.

Whole worm mounted lectin staining was performed as previously described by Borgonie and Driessche [21], with some modifications. Briefly, *C. elegans* were rinsed from NGM culture plates with 15 mls of M9 buffer by slow swirling and this was repeated two times. The volumes were combined and allowed to stand at room temperature for 1 h to allow digestion and expulsion of intestinal contents. After three washes with M9 buffer 1 ml of ice cold acetone was added to the worms and the solution was placed on ice for 2 minutes. *C. elegans* were rehydrated with sequentially washing with 80, 60, 40 and 20% acetone and finally with PBS. Then 50 µl of 10 µg/ml of FITC or Texas Red conjugated lectins suspended in PBS, 0.5% triton X-100 and 0.05% NaN_3_ was added and the slurry was incubated at 4°C for 12 h with gentle rocking. To 10 µl of the stained worm solution was added 10 µl of 1 mg/ml of paraphenylene diamine in Citifluor solution (Sigma) then mounted on washed and dried poly L-lysine slides. FITC conjugated *Ulex eropaeus I* (UEAI), *Triticum vulgare* (WGA), *Agaricus bisporus* (ABA), *Anguilla anguilla* (AAA), *Galanthus nivalis* (GNA) (EY Laboratories) lectins were used in this study except for experiments depicted in Figure 1 where an ABA Texas Red conjugate was used. The microscope used was an Axiovert 100 TV (Zeiss, Germany) equipped with iVision software (BioVision Technology). Percent staining for ABA lectin was performed as follows: One hundred to one hundred and twenty individuals each of N2 and *bus-4* nematodes were observed and scored based on diminished staining in the triangular region emanating from the anus to the tail region. See Figure S1 in supporting information for an example of this region.

### Lectin staining of live nematodes

For each strain, L4 and young adult worms were washed from NGM plates with M9 buffer and allowed to settle to the bottom of a 1.5 mL tube. Settled worms (50 µL) were mixed with 10 µL Texas Red labeled ABA or FITC labeled UEA-1 (1mg/ml), 50 µL of E. coli (200 mg/ml) and 140 µL M9 buffer and rocked in the dark at room temperature for 24 hours in a Costar cluster plate (24 well size). After 24 hours, the samples were removed from the wells and washed two times with M9 buffer by centrifugation (300 × g for 1 min). For shorter ABA staining experiments, L4 and young adult worms were washed from NGM plates and allowed to settle to the bottom of the tubes. Three microliters of worms were mixed with 0.5µl ABA lectin (1 mg/ml), 6.5µl 500 mM GalNAc, and 15 µl M9 buffer and rocked at room temperature. For all staining experiments pelleted worms (10 µL) were mixed with 15 µl of sodium azide (20 mM) on a glass slide, covered with a glass coverslip, and photographed with an Olympus SX16 fluorescence microscope equipped with a DP72 camera. Camera settings were the same for all samples. ImageJ [22] was used to measure the mean fluorescence intensity of the head prior to the metacorpus, the tail beyond the anus, and a 3 pixel (2 µmeter) line through the center of the intestine beginning after the terminal bulb and ending prior to the rectum.

### ABA lectin binding of C. elegans glycopeptides

1.5 mg of *C. elegans* protein was reduced with dithiothreitol, alkylated with iodoacetamide and trypsinized with TPCK trypsin in phosphate buffer (pH 7.5) at 1∶50 trypsin to protein ratio overnight at 37 °C. The digested protein, in 70% acetonitrile 0.1% trifluoroacetic acid, was then applied to a 400 mg Amide-80 (Tosoh, Japan) column equilibrated to 80% acetonitrile 0.1% trifluoroacetic acid. The column was then washed extensively with 5 one milliliter volumes of 80% acetonitrile 0.1% trifluoroacetic acid. Glycopeptides were eluted sequentially with one milliliter each of 60% acetonitrile 0.1% trifluoroacetic acid, and 40% acetonitrile 0.1% trifluoroacetic acid. The fractions were combined, vacuum rotary evaporated and the glycan assessed using the phenol sulfuric assay for neutral hexose. An aliquot was removed for control and the remainder of sample applied to a one milliliter column of ABA lectin (EY Laboratories). The sample was allowed to incubate in the column bed for 30 minutes. Five one milliliter volumes of 10 mM phosphate buffer (pH 7.5) were applied to the column and the run through collected. A one milliliter volume of 100 mM β-methyl galactoside was applied to the column and incubated for 30 minutes. Two more volumes of the β-methyl galactoside solution were added to the column and the eluate collected. Control, run through and eluate fractions were vacuum rotary evaporated and subjected to β-elimination as previously described. The released *O*-glycans from each fraction were permethylated and analyzed by MALDI-TOF MS.

## Results

### bus-4 contains conserved domains of core-1β1,3 galactosyltransferases

The *bus-4* gene, designated as T22B11.2 (WB gene ID WBGene00020676), is located on chromosome IV. A BLAST search of the NCBI database using the predicted 368 amino acid *Bus-4* protein showed it to have a high degree of conservation (expect value: 7e-8) with Pfam01762, a predicted galactosyltransferase family containing a high number of UDP-Gal:β-GlcNAc β1,3- and UDP-Gal: β-GalNAc β1,3- galactosyltranferase (β3-Gal-T) homologs. *Bus*-*4* has 29–35% identity with putative core1 β3-Gal-T enzymes such as the Zebra fish core1 β3-Gal-T (Gene ID 5577675) homolog. They share 35% identity with an expect value of 1e-29. It is also closely related to *C. elegans* gene C38H2.2 (BLAST score 2e-34 relative to *bus-4*) [8], which is an ortholog of human T-synthase and has demonstrated core 1 *O*-glycan synthetic activity by catalyzing the addition of Gal to GalNAcα1-Ser/Thr glycopeptide in vitro [23]. *BUS-4* and the members of this family share the conserved motifs VK*X*TW, G*X*GYV(I)*X*S, and DL*XX*G which have been reported in the human core 1 β 1,3-galactosyltransferase (T-synthase) [24] and are conserved in this transferase family. Additionally *BUS-4* contains a DXD motif, which is conserved in 13 glycosyltransferase families [25]. While these data cannot predict enzymatic activity they do provide evidence that *bus-4* encodes a glycosyltransferase that may be active in core-1 *O*-glycan synthesis.

### Lectin studies in fixed and live nematodes

A panel of lectins was used to characterize the cellular surfaces and surface coat of the N2 and *bus-4* nematodes. Both surface coat and cell surface glycoconjugates interact with the external environment. As the majority of glycosylation active *bus* mutants are expressed in tissues that support the cuticle, intestine, and surface coat our rational was to characterize these regions for gross glycoconjugate expression. The surface coat servers as a barrier to predation and, therefore, any changes could contribute towards the observed bacterial resistance. Likewise, glycoconjugates at the cuticle, intestine, including the anus and rectum, may be involved in surface recognition or delivering cues to bacterial pathogens. These studies allowed us to characterize and better localize glycomics changes, which were further investigated through chemical structure analysis.

#### Fixed nematodes

A panel of lectins was used to examine the characteristics of *C. elegans* cell surfaces. Fluorescent staining of whole mounted acetone-fixed nematodes was performed with the following FITC or Texas Red conjugated lectins: Galβ1,3-GalNAc specific, *Agaricus bisporus* (ABA), α-L-fucose specific *Ulex europaeus I* (UEA I) and *Anguilla anguilla* (AAA), β1,4-GlcNAc specific *Triticum vulgare* (WGA), and Manα1-3,6 specific *Galanthus nivalis* (GNA). The specificity of each lectin was verified by preincubation of the lectins with an inhibitory concentration of the corresponding sugar prior to incubation with the *C. elegans* strains. A minimum of 100 nematodes per strain were examined in each experiment. Mannose specific GNA lectin staining of the cuticle surface was appreciably brighter over the central and distal portion of the cuticle of the *bus-4* strain (Figure S17). UEA I (α-L-fucose) staining in the tail region and intestine of the *bus-4* mutant was also appreciably brighter (Figure S18). Little or no differences were observed with, WGA (GlcNAc), or AAA (α-L-fucose) lectin staining (Figures S19 and S20).

There was a striking difference in Galβ1,3GalNAc ABA lectin staining as shown in Figure 1. This lectin detects the above core-1 disaccharide in mammalian samples and *Ce* core-I *O*-glycans in *C. elegans* as previously described [13]. The fluorescence intensity was much lower in the *bus*-*4* mutant over the surface of the acetone fixed cuticle. This was especially so in the tail region indicating that the *Ce* core-I *O*-glycan epitopes were diminished at the cuticle surface and notably so in the region that *M. nematophilum* infects in wild type nematodes (see Figure S1). This pattern was similar to that observed in *bus-2* nematodes [13]. Reduced ABA staining intensity was observed in greater than 90% of observed *bus-4* nematodes. Staining was more intense in some *bus-4* juveniles but never approached the intensity seen in the wild type counterparts. Glycolipid is not a likely source of signal as these preparations were delipidated through the acetone fixation process. To confirm that the loss of ABA staining in the *bus-4* background was *bus-4* dependent a wild type *bus-4* transgene was introduced and the rescued strain was compared to both N2 wild type and *bus-4* deficient strains as shown in Figure 1. The rescued strain (CB6817) regained ABA staining over the cuticle surface and the staining was especially intense in the tail region, a pattern that was nearly identical to that of the parent N2 strain.

#### Live nemotodes

**Figure 2:**
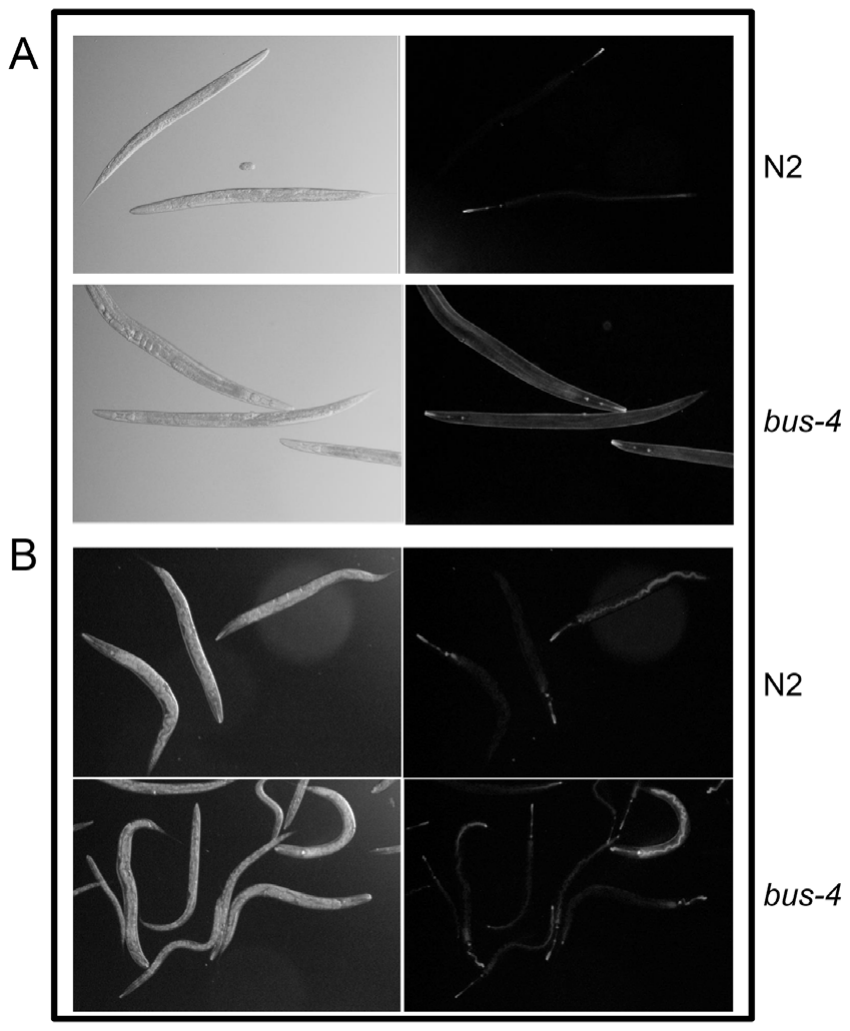
Lectin staining patterns of live *bus-4* nematodes are altered. N2 and bus-4 nematodes were stained with FITC conjugated *Agaricus bisporus* (ABA) Gal (β1,3) GalNAc specific (panel A) and *Ulex europaeus* (UEA-I) α-L-Fuc specific (panel B) lectins. ABA staining in *bus-4* is more intense over the surface coat and most dramatic in the head and tail regions. UEA-I staining in *bus-4* is more intense in the hind gut, anus and rectum.

When we determined the amount of *N*- and *O*-glycans released from *bus-4* and N2 Bristol nematodes we found that the amount of *N*-glycans released per gram of worms was nearly equivalent for the two strains (N2  =  146 µg/g; bus-4  =  156 µg/g). However, the *bus-4 O*-glycans were twice as abundant as those from N2 Bristol (N2  =  1.2 mg/g; *bus-4*  =  2.4 mg/g). The lectin studies described previously were performed under acetone fixed conditions in the absence of cross-linking reagents, allowing removal of soluble components. To assess glycoproteins of the surface coat and other soluble components that could contain *Ce* core-I glycoproteins, such as mucin-like proteins, we performed ABA lectin staining studies of live nematodes. As shown in Figure 2A, ABA stains the entire surface coat more strongly in live *bus-4* nematodes compared to N2 nematodes, and especially so in the head and tail regions. Head region average pixel intensity for N2 was 24.1 ± 4.1 SD and that for *bus-4* was 52.7 ± 10.1 SD. Tail region average pixel intensity was 10.4 ± 4.4 SD for N2 and 25.5 ± 12.0 SD for *bus-4* respectively. Staining was more than twice as intense in both of these areas in the *bus-4* strain. The average pixel intensity in the intestine of nematodes fasted for one hour was also slightly increased but the difference was not statistically significant. Staining was also seen in both strains in the region of the pharynx and most prominently in the posterior bulb. These data show that *C. elegans bus-4* mucin glycoprotein abundance and distribution are dramatically altered. However, it cannot be ruled out that some staining intensity may be contributed from glycolipid.

Live N2 and *bus-4* nematodes were stained with UEA-I as was performed for ABA as shown in Figure 2B. While in both fasted strains staining was seen in the head, pharynx and intestine, in *bus-4* staining was concentrated in the distal intestine and stained densely in the anus and rectal region. In some instances *bus-4* nematodes stained 3–4 times more intensely compared to N2 counterparts. However, staining was not consistent as ABA staining patterns and the lectin appeared to have a toxic effect leading to decreased feeding and mobility which was more pronounced in some experiments.

### ABA lectin binds C. elegans O-glycopeptides

To test the specificity of the ABA lectin in *C. elegans* N2 Bristol and *bus-4* glycopeptide extracts were subjected to ABA lectin affinity column analysis. Glycoprotein extracts were reduced, alklyated with iodoacetamide, and trypsinized. The mixture was subjected to amide 80 solid phase extraction yielding eluate enriched in *C. elegans* hydrophilic glycopeptides. Glycopeptides were used rather than β-eliminated free glycans as the former are known to have higher binding affinity to this lectin [26]. The enriched fractions were then applied to ABA lectin columns, washed extensively with 10 mM phosphate buffer, and the bound species eluted using 100 mM β-methyl galactoside. Control (Amide-80 eluted glycopeptide), ABA column run through, and eluate were dried and subjected to β-elimination as previously described (see Materials and Methods). Glycans released by β-elimination were polished with porous graphite solid phase extraction columns and permethylated. MALDI-TOF MS experiments were performed on all fractions.

MALDI-TOF MS permethylated glycan profiles from control sample, ABA flow through, and elution fractions for the N2 and *bus-4* derived glycans are shown in Figure S2. Control is shown in panels A and D (β-eliminated Amide 80 enriched fraction), the flow through in the center panels B and E, and the β-methyl galactoside eluated fractions in panels C and F. The control shows the presence of the major *Ce* core-I *O*-glycans (Hex_3_HexNAc, Hex_4_HexNAc, and HexA_1_Hex_3_HexNAc are shown), and some polyhexose (Hex_5_ and Hex_6_ are shown) a common *C. elegans* glycan preparation contaminant. The flow through contained primarily the polyhexose glycans. The eluate from both strains containing glycans that bound to the ABA lectin, included the major *Ce* core-I *O-*glycans Hex_3-4_HexNAc_1_, and HexA_1_Hex_3_HexNAc. These data demonstrate that the ABA lectin recognizes the *Ce* core-I *O-*glycans from both N2 and *bus-4* nematodes.

### Analysis of O-glycans

**Figure 3:**
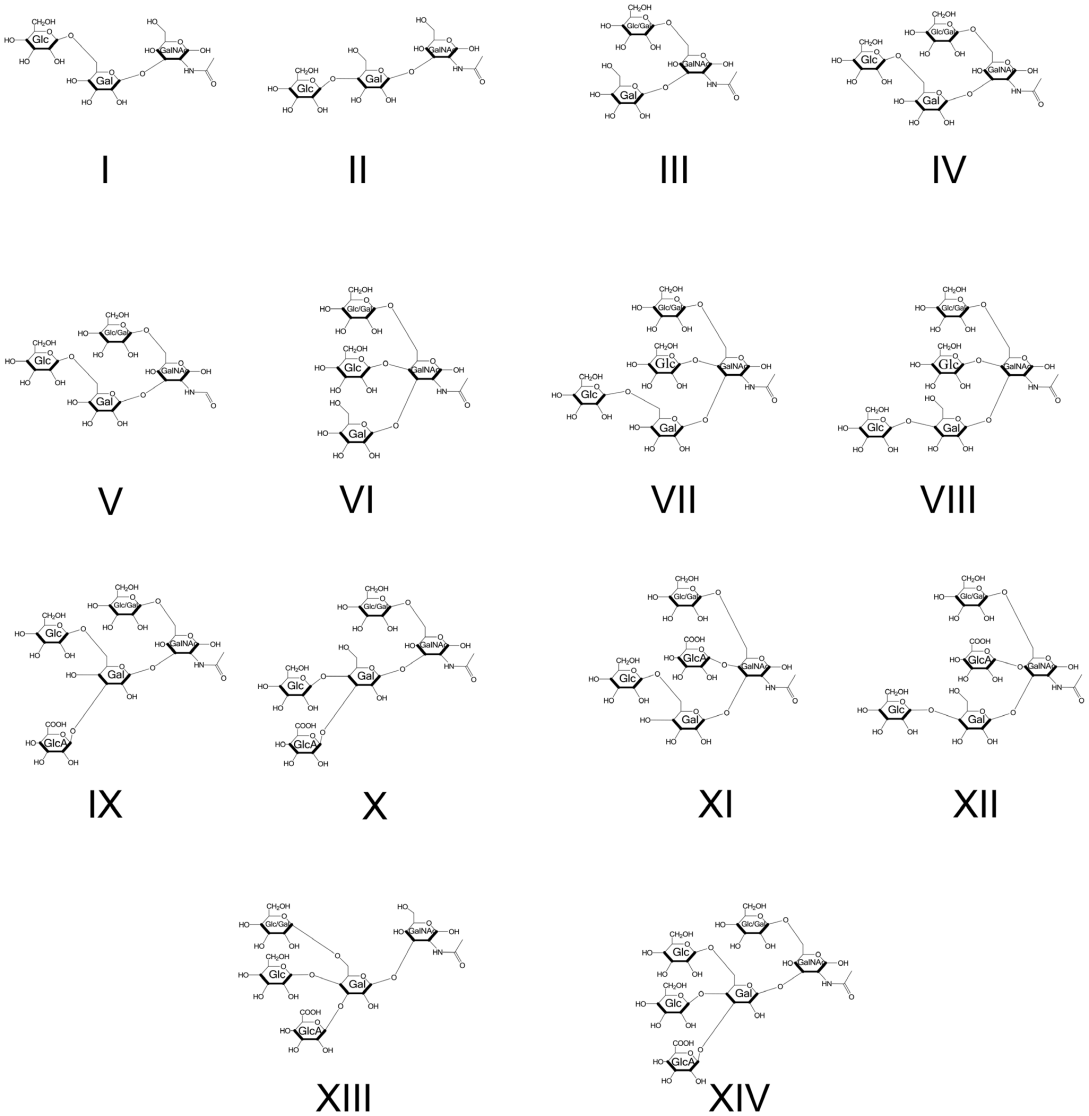
The structural configurations of the *Ce* core-I *O*-glycans defined in this study are shown.

*O*-glycans were released by β-elimination, permethylated and analyzed by MALDI-TOF MS. The *bus-4 O*-glycan compositions detected in this study are shown in Table 1 and select ones in Figure 3. The *O*-glycans of *C. elegans* can be placed into four sub-groups, (i) neutral *Ce* core-I, (ii) acidic *Ce* core-I, (iii) fucosylated neutral, and (iv) *Ce* core-II (also highly fucosylated). These forms have been previously described [14], [27]–[29].

**Table 1:** Composition of *C. elegans* permethylated *O*-glycans released from N2 wild type and the mutant strain *bus*-*4* determined by MALDI-TOF MS analysis.

Measured *m/z*	Calculated *m/z*	Composition^a^
534.15	534.29	Hex1HexNAc_1_
708.32	708.38	dHex_1_Hex_1_HexNAc_1_
738.36	738.39	Hex_2_HexNAc_1_
752.34	752.40	Hex_1_HexNAc_1_HexA_1_
912.51	912.48	dHex_1_Hex_2_HexNAc_1_
926.43	926.49	dHex_1_Hex_1_HexNAc_1_HexA_1_
942.53	942.49	Hex_3_HexNAc_1_
956.51	956.50	Hex_2_HexNAc_1_HexA_1_
1116.66	1116.58	dHex_1_Hex_3_HexNAc_1_
1130.51	1130.59	dHex_1_Hex_2_HexNAc_1_HexA_1_
1146.68	1146.59	Hex_4_HexNAc_1_
1160.66	1160.60	Hex_3_HexNAc_1_HexA_1_
1320.77	1320.68	dHex_1_Hex_4_HexNAc_1_
1332.72	1331.69	dHex_2_Hex_2_HexNAc_2_
1334.78	1334.69	dHex_1_Hex_3_HexNAc_1_HexA_1_
1345.74	1345.71	dHex2Hex_1_HexNAc_2_HexA_1_
1364.71	1364.70	Hex_4_HexNAc_1_HexA_1_
1375.73	1375.72	dHex_1_Hex_2_HexNAc_2_HexA_1_
1494.88	1494.77	dHex_2_Hex_4_HexNAc_1_
1549.92	1549.81	dHex_2_Hex_2_HexNAc_2_HexA_1_
1579.97	1579.82	dHex_1_Hex_3_HexNAc_2_HexA_1_
1699.01	1698.87	dHex_2_Hex_5_HexNAc_1_
1724.03	1723.90	dHex_3_Hex_2_HexNAc_2_HexA_1_
1928.12	1928.00	dHex_3_Hex_3_HexNAc_2_HexA_1_
2102.22	2102.09	dHex_4_Hex_3_HexNAc_2_HexA_1_
2306.98	2306.19	dHex_4_Hex_4_HexNAc_2_HexA_1_
2480.73	2480.28	dHex_5_Hex_4_HexNAc_2_HexA_1_
2510.38	2510.29	dHex_4_Hex_5_HexNAc_2_HexA_1_
2685.12	2684.38	dHex_5_Hex_5_HexNAc_2_HexA_1_

a All ion compositions detected as sodium adducts

**Figure 4:**
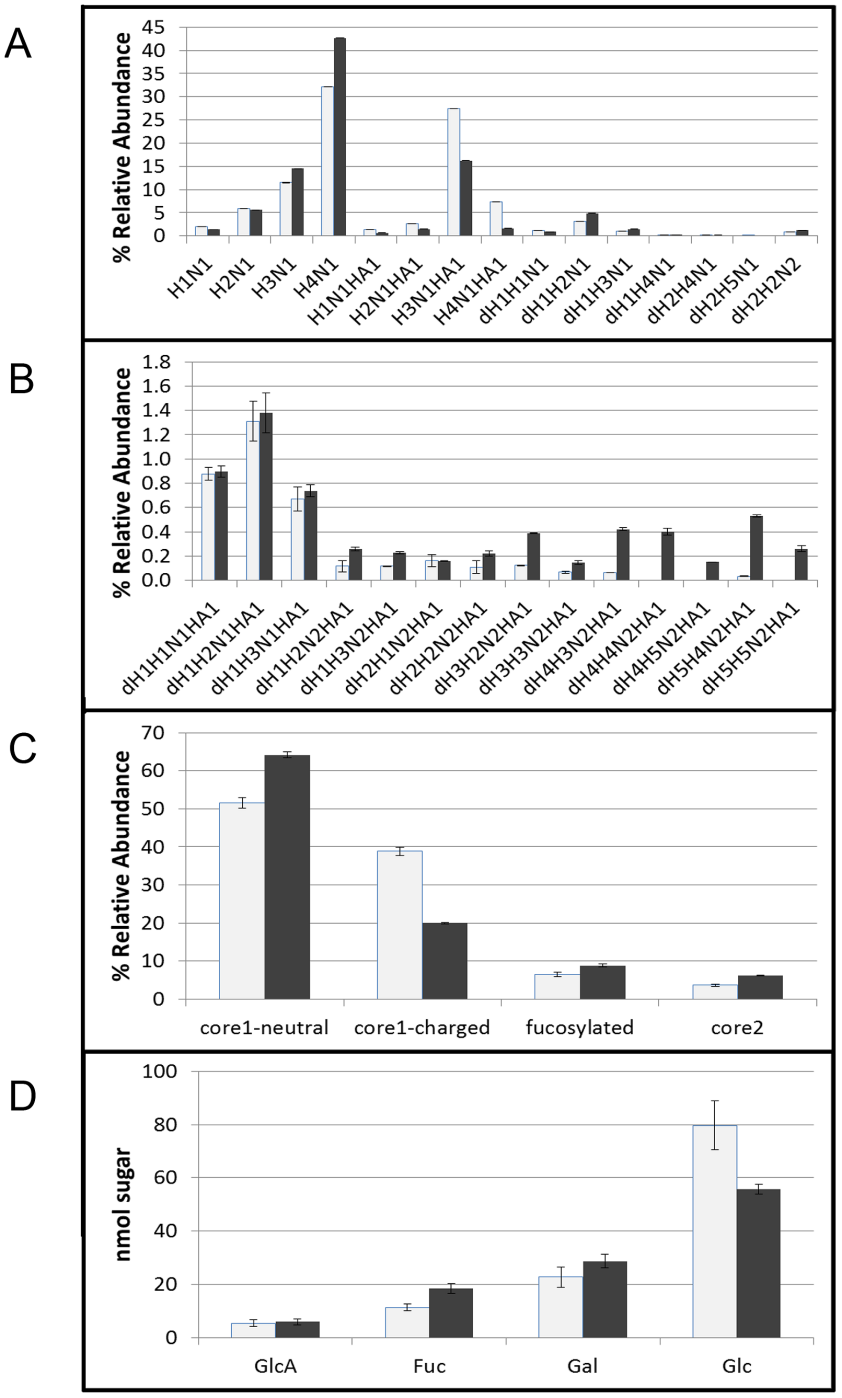
MALDI-TOF MS permethylation profiling of N2 and *bus-4* O-glycans. Histograms represent the average of three independent analyses. Light gray histograns are N2 and black are *bus-4*. Sample spectra are shown in Figure S3. Error bars represent one standard deviation. A: Relative quantitation of Ce core-I neutral, Ce core-I charged and fucosyl glycoforms, B; Relative quantitation of Ce core-II glycoforms, C; Relative abundances of *O*-glycan subclasses, D; GC/MS monosaccharide abundances detected in this study

Histograms of glycan compositions produced from three MALDI-TOF MS replicates are shown in Figure 4A (*Ce* core-I neutral and charged, and fucosyl) and Figure 4B (*Ce* core-II). The combined intensities in each subclass are shown in Figure 4C. Error bars express 1 SD. Representative spectra are shown in Figure S3. Relative percentages of wild type *C. elegans Ce* core-I neutral glycans were 52%, *Ce* core-I charged forms 39%, neutral fucosyl forms 7%, and *Ce* core-II glycans less than 4% of the total *O*-glycans. In *bus-4* nematodes higher levels of *Ce* core-I neutral, 63% (↑25%), fucosylated neutral, 9% (↑28%), and *Ce* core-2, 7% (↑60%) were detected. Lower levels of the *Ce* core-I charged *O*-glycans, 20% (↓52%) were observed.

### GC/MS analysis of C. elegans O-glycans

N2 and *bus-4 O*-glycan monosaccharides were analyzed in triplicate by GC/MS as their trimethylsilyl derivatives. Comparisons of monosaccharide abundances are shown in Figure 4D. Compared to monosaccharides derived from N2 *O*-glycans, those from *bus-4* nematodes contained significantly lower amounts of Glc (↓31%) and higher amounts of Gal (↑26%) and Fuc (↑56%). GlcA abundances in the two *O*-glycan pools were similar. Noting that larger glycans ionize more efficiently than smaller ones in MALDI-TOF MS analysis, this result in the *bus-4* monosaccharide profile is likely reflective of a counter balancing between an increase in *Ce* core-II and decrease in *Ce* core-I charged glycans, both of which contain GlcA. Increases in *bus-4* fucosyl glycoforms, the neutral fucosyl and *Ce* core-II *O*-glycans are also reflected in the monosaccharide analysis results. Low abundance GlcNAc, Man and Xyl peaks were also detected likely from residual high mannose *N*-glycans for the former and trace glycosaminoglycans for the later due to imperfect enzymatic release and purification respectively. Core GalNAc-ol was detected only in trace amounts as the sugar alcohols are less stable than reducing sugars under the conditions of analysis used here.

### Tandem MS analysis of permethylated O-glycan isomers

**Figure 5:**
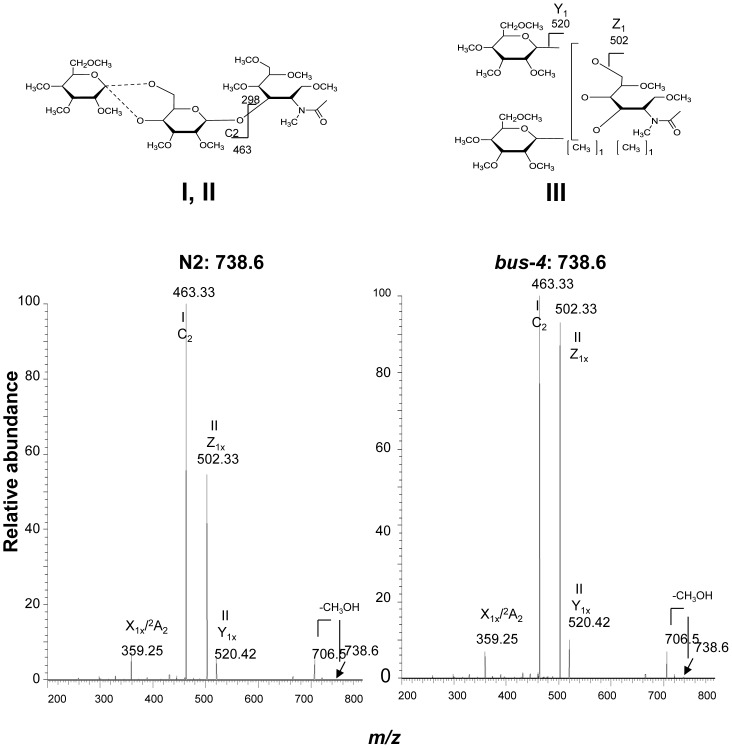
The CID MS^2^ analysis of matched N2 and *bus-4* permethylated Hex_2_HexNAc_1_-ol at *m/z* 738.6 [M+Na]^+^. Data were collected under identical conditions using a Thermo LTQ-XL ion trap equipped with an Advion Nanomate sample infusion system. Three predicted isomeric structures (I, II and III) are represented. The origin of key fragment ions are shown. Structures I and II differ only by the position of the substituent Glc at either C4 or C6 of the partner Gal residue as indicated by the dashed line. See Figure 2 for inferred monosaccharide identities.

**Figure 6:**
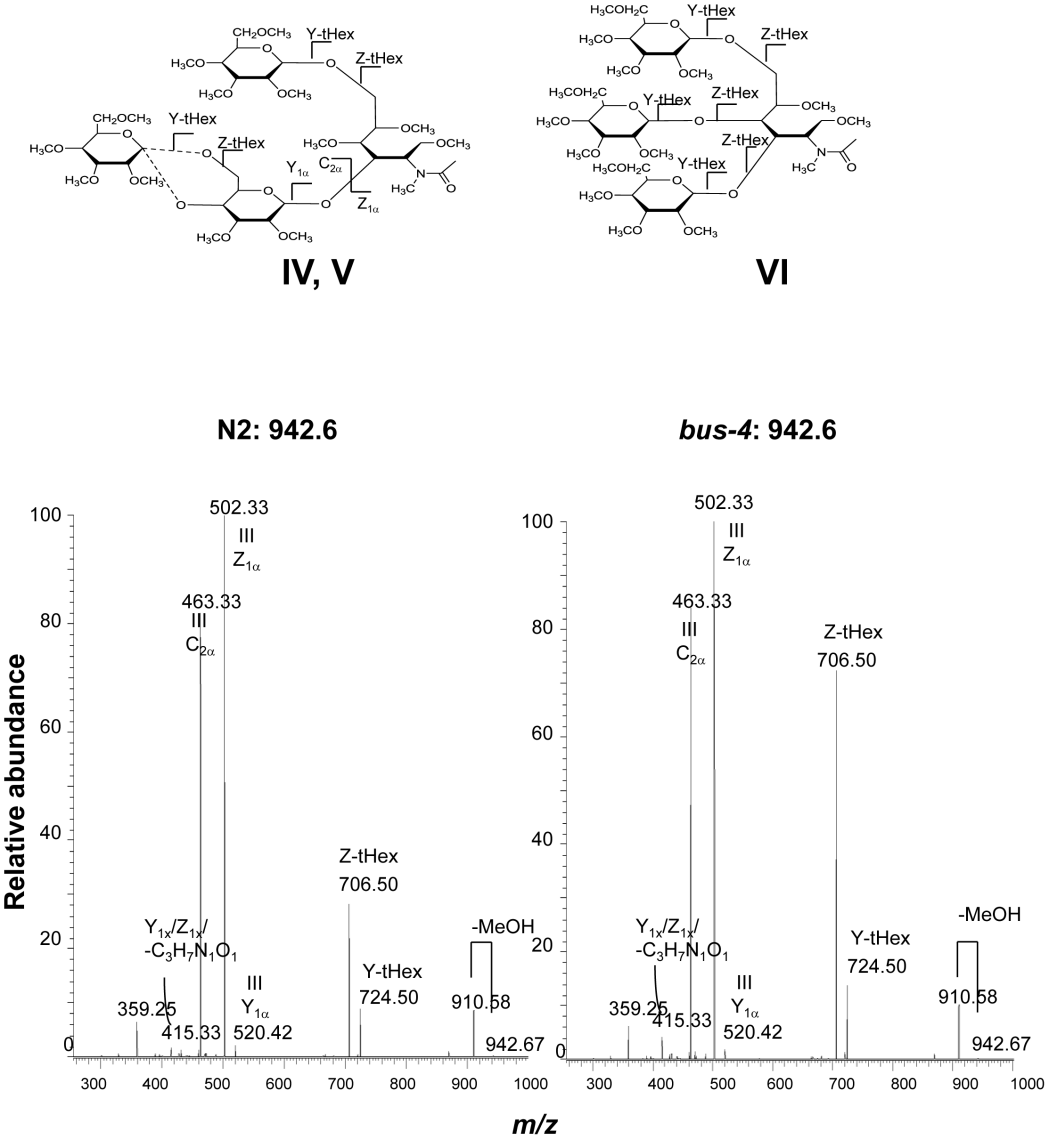
The CID MS^2^ analysis of matched N2 and *bus-4* permethylated Hex_3_HexNAc_1_-ol at *m/z* 942.6 [M+Na]^+^. Data were collected under identical conditions using a Thermo LTQ-XL ion trap equipped with an Advion Nanomate sample infusion system. Three predicted isomeric structures (IV, V and VI) are represented. The origin of key fragment ions are shown. Structures IV and V differ only by the position of the substituent Glc at either C4 or C6 of the partner Gal residue as indicated by the dashed line. See Figure 2 for inferred monosaccharide identities.

**Figure 7:**
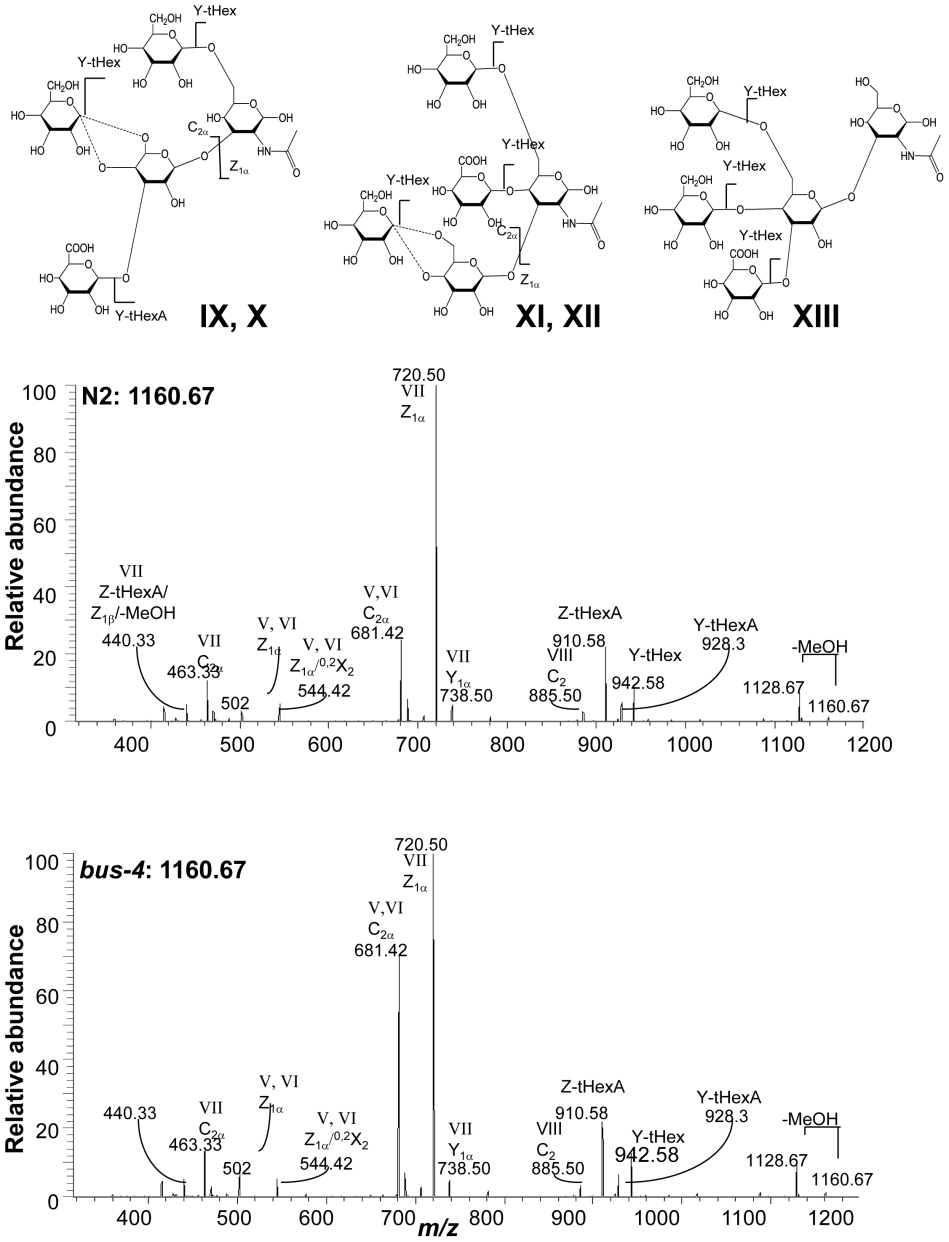
The CID MS^2^ analysis of matched N2 and *bus-4* Hex_3_HexNAc_1_HexA_1_-ol at *m/z* 1160.6 [M+Na]^+^. Data were collected under identical conditions using a Thermo LTQ-XL ion trap equipped with an Advion Nanomate sample infusion system. Five predicted isomeric structures (IX – XIII) are represented. The origin of key fragment ions are shown. Structural pairs IX and X and XI and XII differ only by the position of the substituent Glc at either C4 or C6 of the partner Gal residue as indicated by the dashed line. See Figure 2 for inferred monosaccharide identities.

To further investigate the *O*-glycan pools, comparative tandem MS analysis was performed on matched N2 Bristol and *bus-4* derived glycans samples. Individual compositions were analyzed for differences in isomer distributions. No differences were seen in fragment ion abundances in matched neutral fucosyl or *Ce* core-II glycan isobars. However, fragment ion abundances from matched *Ce* core-I glycan isobars, both neutral and charged forms, differed significantly. Key differences are highlighted below. The derived glycan structures are shown in Figure 3. We add that the *O*-glycan structures derived in the present study are in agreement with those reported in the pioneering work of Guerardel *et al*., [29] and our later investigations [14], [30]. Anomeric configurations and monosaccharide identities are inferred from the Guerardel et al., study as supported by the data contained herein. As the previous studies were not exhaustive, related glycans of intermediate size and configuration can be expected and these were found in the present study. Additionally we found a GlcA substitution that is in addition to the one previously reported, which will be described later. Note that fragment ion peaks are identified using the established standard nomenclature of Domon and Costello [31] in MS^2^ spectra (Figures 5, 6, 7, S9 and S14). The MS^3^ fragment ion peaks shown in spectra in supporting information figures (Figures S4-S8 and S11-S15) are identified numerically based on *m/z* of daughter ions for simplicity to aid in understanding of the structural assignments presented in the figures.

The MS^2^ spectra of the permethylated N2 (left) and *bus-4* (right) derived Hex_2_HexNAc_1_, *m/z* 738.6 [M+Na]^+^, are shown in Figure 5. The fragmentation pattern indicated the presence of three distinct configurations identified as Structures I, II and III in Figures 3 and 5. There was considerably more fragment ion abundance at *m/z* 502.33 in the *bus-4* Hex_2_HexNAc_1_spectrum. The ion abundances at *m/z* 502.33 from both N2 and *bus-4* were subjected to MS^3^ analysis. The spectra are shown in Figure S4. The daughter ion patterns were consistent with a Hex1,6HexNAc- configuration in both the N2 and *bus-4*. The rational for structural assignment is presented in the figure. The MS^3^ spectra from the *m/z* 502.33 daughter ions from each strain contained the same daughter ion *m/z* values but they differed in their abundances. This can occur when there is a difference in monosaccharide components even when linkage configuration is the same [32]. We concluded that there was likely a difference in the ratio of Glcβ1,6 to Galβ1,6 at the core GalNAc residue in the N2 and *bus-4* derived Hex_2_HexNAc_1_ pools. Both of these substitutions have been previously documented [29]. Ion abundances at *m/z* 463.33 were also investigated by MS^3^ analysis (Figure S5). This ion contains the Glcβ1,4Gal and Glcβ1,6Gal portions of Structures I and II. Evidence for both configurations was present. The rational for structural assignment is presented in the figure. In summary, abundances of Structures I and II were lower in the *bus-4* pool. As only Structures I and II contain terminal Glcβ substitution of the core Gal, less of these were present in the *bus*-4 Hex_2_HexNAc_1_ pool. Structure III was more abundant in *bus-4 O*-glycans and the ratio of Glcβ1,6 and Galβ1,6 at the core GalNAc residue was likely altered.

The MS^2^ analysis of N2 and *bus-4* Hex_3_HexNAc_1_, *m/z* 942.6 [M+Na]^+^ are shown in Figure 6. Three structural configurations were detected and are shown as Structures IV, V and VI in Figures 3 and 6. More than twice the ion abundance of the *m/z* 706.5 daughter ion was detected in the *bus-4* glycan spectrum. The MS^3^ fragmentation pattern of *m/z* 706.5 from both glycan sources was identical. The spectra derived structure and rationale are shown in Figure S6. The *m/z* 706.5 ion arises from Structure VI and is produced after loss of the Galβ1,3- arm linked to the core GalNAc. These data show that more of Structure VI is present in the *bus-4 O*-glycan pool. MS^3^ of N2 and *bus-4* *m/z* 502.3 ions produced essentially identical patterns as shown in Figure S7 showing the presence of Hex1,6GalNAc in both strains. The rationale for the structural assignment is shown in the figure. Therefore the ratio of Glcβ1,6GalNAc and Galβ1,6GalNAc moieties in Structure IV and V in the N2 and *bus-4* Hex3HexNAc1 pools were essentially the same. Ion abundance at *m/z* 463.33 was also investigated by MS^3^ analysis (Figure S8). This ion contains the Glcβ1,4Gal and Glcβ1,6Gal component of Structures IV and V. Evidence for both linkage configurations was present. The spectra derived from both strains' glycans were essentially identical. Therefore no differences in the ratio of Glcβ1,4Gal and Glcβ1,6Gal were seen in IV and V. The major difference seen in the *bus-4* Hex_3_HexNAc_1_ pool was an increased abundance of Structure VI and less of Structures IV and V. As a result, less terminal Glcβ substitution of core Gal was present in the *bus-4* Hex_3_HexNAc_1_pool.

The N2 and *bus-4* Hex_4_HexNAc_1_ ion abundances at *m/z* 1146.70 [M+Na]+ were examined by tandem MS. All matched tandem MS spectra from the *bus-4* and N2 derived glycans were essentially identical (Figure S9). The data were consistent with the derived configurations shown as VII and VIII shown in Figure 3. No evidence for a difference in configuration was seen in the Hex_4_HexNAc_1_ pools. However, as shown in Figure 4A, this composition is increased 32% in abundance in the *bus-4 O*-glycan pool as seen by MALDI-TOF MS analysis.

MS analysis of the matched N2 and *bus-4* charged *Ce* core-I glycans also revealed differences in isomer distribution. The spectra resulting from MS^2^ decompositional analysis of HexA_1_Hex_3_HexNAc_1_, *m/z* 1160.7 [M+Na]^+^, (diminished by ∼40%, in *bus-4*) are shown in Figure 7. Based on the MS^2^ and subsequent MS^3^ decompositional analysis there were five configurations present as shown in Figure 3 and 7 as Structures IX through XIII. More ion abundance was seen at *m/z* 681.42 in the *bus-4* HexA_1_Hex_3_HexNAc_1_spectrum. This ion abundance can only arise from Structures IX and X. MS^3^ comparisons of this fragment derived from the N2 and *bus-4* are shown in Figure S10. Note in this figure that the position of GlcA substitution in Structures IX and X is defined at C3 of the Core Gal in agreement with that reported previously [29].The spectra were virtually identical indicating that the Glcβ1,4(GlcAβ1,3)Galβ- and Glcβ1,6(GlcAβ1,3)Galβ- portions of IX and X were the same in N2 and *bus-4* even though the two configurations were proportionately increased in the *bus-4* HexA_1_Hex_3_HexNAc_1_ pool. The *m/z* 502.3 daughter ion in Figure 7 was substantially increased in abundance in the *bus-4* glycan spectra as well as it also can only arise from IX and X. MS^3^ of the matched pair verified it to arise from the Hex1,6GalNAc portion of Structures IX and X (Figure S11). However the spectra were different in daughter ion abundances showing that the ratio of Glcβ1,6/Galβ1,6 substitutions at the core GalNAc were likely different in IX and X between the two strains as seen previously for Structure III. The presence of Structures XI and XII were confirmed by MS^3^ analysis of ion abundances at *m/z* 720.5, which identified the Hex1,6(Glc1,4)GalNAc moiety (see Figure S12). The presence of Structure XIII was confirmed with MS3 fragmentation of *m/z* 885.5. The spectra and rational can be seen in Figure S13. Ions specific for Structure XIII were low in abundance in both strain derived pools. Overall in *bus-4* Structures IX and X were increased, XI and XII were decreased and the amount of XIII was similar compared to those of N2. Ratios of Glcβ1,6/Galβ1,6 substitutions at the core GalNAc likely differed and, based on MALDI-TOF MS analysis, the HexA_1_Hex_3_HexNAc_1_ pool was diminished by 40% in the *bus-4 O*-glycan pool (see Figure 4A). Structures IX and X, and XI and XII differ in the position of GlcA substitution. The former two, Structures IX and X, are GlcA substituted at C3 of the core Gal whilst Structures XI and XII are GlcA substituted at C4 of GalNAc. These changes shift the diminished HexA_1_Hex_3_HexNAc_1_ pool strongly toward the GlcAβ1,3Gal substitution. We note that the GlcA substitution at C4 of core GalNAc has not been previously reported. See Figure S12 for the rational of structural assignment.

The N2 and *bus-4* derived HexA_1_Hex_4_HexNAc_1_ MS^2^, *m/z* 1364.8 [M+Na]+, fragmentation patterns were essentially identical (Figure S14). This composition was diminished ∼80% in the *bus-4 O*-glycan pool as shown in Figure 4. We investigated the *m/z* 502.4 fragment ion abundance for evidence of Glc1,6-/Gal1,6- GalNAc substitution distribution shift. Again we observed differences in this fragment's daughter ion abundances consistent with a shift in Glcβ1,6-/Galβ1,6-GalNAc substitution ratio (Figure S15). The major differences in the *bus-4* HexA_1_Hex_4_HexNAc_1_ composition was an altered presumptive ratio of Glcβ1,6-/Galβ1,6 on core GalNAc and an 80% decrease in relative abundance of this composition in the *bus-4* pool.

The abundances of specific structural components in the *bus-4* and N2 *Ce* core-I *O*-glycan pools were compared. The apparent % difference of each structural component was calculated based on *Ce* core-I pool composition abundances from quantitative MALDI-TOF MS, and key isomer fragment ion abundances, from ion trap decompositional analysis, (see Material and Methods). Results of this comparison are shown in Table 2. Differences in *bus-4* relative to N2 (reported to 1SD) were as follows: Hex1,4 substitution of core GalNAc was increased 38.5% (±5.14), GlcA1,4 substitution of the core GalNAc was decreased 42.2% (±12.2) and GlcAβ1,3 substitution of core Gal was decreased by 30% (±22.7). Glcβ1-4,6 terminal substitutions of core Gal were decreased 5.8% (±4.7), Glcβ1,6/Galβ1,6 substitution of core GalNAc was increased 7.0% (±4.9).

**Table 2:** Substitution ratios of *bus-4 Ce* core-I glycans differ from wild type.

Substituent Ratio Type	N2 Substituent Ratio (mol/mol core-I saccharide)^a^^,^ b	bus-4 Substituent Ratio (mol/mol core-I saccharide)^a^^,^ b	% Difference relative to N2^c^
Glcβ1-4,6Gal	1.02 (±0.024)	0.89 (±0.023)	−5.8 (±4.7)
Glc/Galβ1-6GalNAc	0.92(±0.029)	0.95 (±0.021)	7.03 (±4.9)
Glcβ1,4GalNAc	0.45 (±0.010)	0.64 (±0.011)	38.5 (±5.14)
GlcA1,4GalNAc	0.2203 (±0.004)	0.16 (±0.004)	−42.2 (±12.2)
GlcAβ1,3Gal	0.1815 (±0.015)	0.075 (±0.015)	−30.0 (±22.7)

a Total molar ratios differ slightly from those reported in Figure 2 as these are calculated only on Ce core-I glycans examined through exhaustive MS^n^ and not the total *O*-glycan pool as seen in Figure 2.

b Figures present in parentheses represent 1SD.

c Figures in parentheses represent 1SD expressed in %.

Overall the *bus-4 Ce* core-I *O*-glycan pool is dramatically altered. The *bus-4 Ce* core-I *O*-glycans' charged residues were reduced by half compared to the N2 parent strain. The remaining GlcA structural distribution is shifted away from C4 of core GalNAc and towards C3 of core Gal. The terminal substitutions are also shifted. The amount of *bus-4 O*-glycan material was doubled compared to the parent strain when measured as milligrams of saccharide per gram wet weight of nematode material The *bus*-4 strain yielded 2.39 mg/g versus N2 which yielded 1.17 mg/g.

### Analysis of N-glycans

*C. elegans N*-glycans were released sequentially using PNGase F followed by PNGase A. PNGase F will not release glycans modified with Fucα1,3 substituted reducing end GlcNAc while PNGase A will do so [33], [34]. Using this sequential release strategy it is our experience that the majority of *N*-glycans are released by PNGase F while a minority are released by subsequent PNGase A treatment. Performed in this way the PNGase A pool can be enriched in fucosyl forms (see Figure S16). Glycans were permethylated and analyzed in triplicate by MALDI-TOF MS. The *bus-4* glycans were compared with those of N2 Bristol. A list of the *N*-glycan compositions detected in this study are shown in Table S1.

The *bus-4* and N2 *N*-glycan PNGase F released pools MALDI-TOF MS relative abundances were examined. High mannose and pauci mannose glycoforms were increased ∼10% and ∼4% of total abundance respectively in the *bus-4* derived pool (Figure S15A) consistent with increased cuticle GNA lectin staining patterns observed in acetone fixed *bus-4* nematodes. There was a decreased abundance of Hex_4-7_HexNAc_4_ mammalian-like and fucosyl glycoforms in the *bus-4* sample PNGase F pool. (see Figure S15B and Figure S21). It should be noted that the majority of fucosyl glycoforms are released in the subsequent PNGase A pool. We further analyzed the PNGase F *N*-glycans as 2-aminobenzamide derivatives by LC/MS ion trap analysis. No additional differences were detected in any glycoforms including isomer distributions.

The PNGase A released pool of *bus-4* contained a 15% higher relative abundance of fucosyl *N*-glycans relative to N2 as shown in Figure S16 and S22. Fourteen fucosyl glycan compositions were detected (see Table S1). Among those, eleven were between 3-50% more abundant in the *bus-4* pool. As PNGase A and not PNGase F releases Fucα1,3 core substituted *N*-glycoforms this was considered significant. A small amount of high mannose and complex (mammalian-like) glycans were also released due to incomplete release by PNGase F. As the PNGase A pool contained only 10% of total *N*-glycans these small differences were not considered significant.

### Glycomics expression analysis

**Figure 8:**
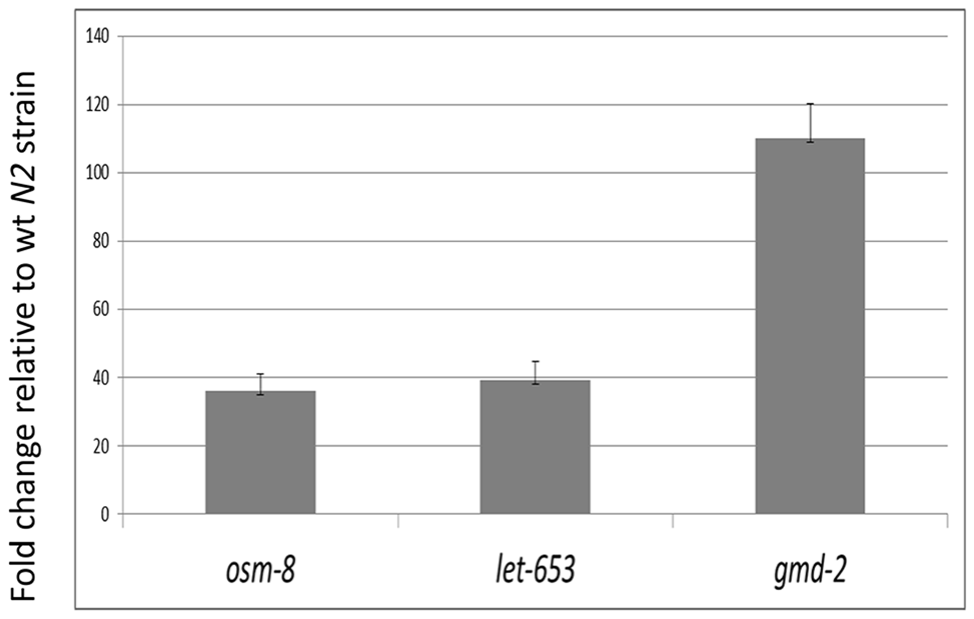
Quantitative RT-PCR analysis *osm-8*, *let-653* and *gmd-2* gene expression: Larval stage L4 of C. elegans larval stage 4 wt (N2) and bus-4 nematodes were analyzed as biological triplicates. Data were presented as relative ΔCts values and normalized to the N2 samples. Error bars represent ± SEMs.

qPCR analysis was performed on key enzyme and glycoprotein transcripts involved in glycoconjugate synthetic pathways Transcripts of *gmd-2*, *osm-8* and *let-653*, *bus-2*, *bus-8*, *gly-11* and *lipl-1* were analyzed (see Materials and Methods). In comparison to N2 only gmd-2, osm-8 and let-653 were greatly upregulated whilst bus-2 (2.1 fold), bus-8 (2.6 fold), gly-11 (1.5 fold), and lipl-1 (2.0 fold), were essentially unaltered or only mildly upregulated. Figure 8 shows the fold change expression in key upregulated genes. The *bus-4* GDP-Man 3,5 dehydratase expression was dramatically increased 110 ± 10 fold. This enzyme catalyzes the committed step in conversion of GDP-Man to GDP-Fuc, the donor substrate in the synthesis of fucosyl glycoconjugates These data are consistent with the increases in fucosyl glycoforms observed in both the *N*- and *O*-glycan pools. Also increased were *osm-8* and *let-653*, two key mucins in *C. elegans*. Both of these were increased 36 ± 4 and 39 ± 4 fold.

## Discussion

The *bus-4* strains were isolated in genetic screens for altered susceptibilities to *M. nematophilum*, *Y. pestis* and *Y. peudotuberculosis*. The *bus-4* gene shows strong conservation with the galactosyltransferase Pfam01762, which contains a high number of UDP-Gal:β GlcNAc β1,3 and UDP-Gal: βGalNAc β1,3 galatosyltransferases, active respectively in the synthesis of the lacto series oligosaccharides types 1 and 2, and Gal β1,3GalNAc core-1 and core-2 *O*-glycans [8]. Studies of the predicted protein sequence strongly suggests that *BUS-4* is a glycosyltransferase that may have a similar activity to members of this glycosyltransferase family.

We have compared the protein glycosylation profile of the *bus-4* reference strain to that of its parent strain N2 Bristol. The most dramatic changes were seen in the *Ce* core-I *O*-glycans which contain the conserved Gal β1,3GalNAc core seen in higher eukaryote core-1 and core-2 *O*-glycans [35]. *C. elegans Ce* core-I glycans differ from the mammalian counterparts significantly. In mammals *O*-glycans are capped with sulfate, sialic acids and Fuc or can be elongated such as with LacNAc extensions and blood group class modifications [35], [36]. In *C. elegans Ce* core-I glycans there is little evidence of extension. Rather, the Gal β1,3GalNAc core can be decorated with terminal substitutions of Glc, Gal, Fuc and GlcA. Rather than sialic acids or sulfate, *C. elegans* uses GlcA as its charge group. No evidence of sulfation of any of its *O*-glycans has been reported except for the heparans [37]. In the present study the *Ce* core-I glycans are shown to be the *C. elegans* mucin *O*-glycans and in the *bus-4* mutants these are dramatically altered as was the expression of two key mucins glycoproteins, *let-653* and *osm-8*.

We observed greatly altered mucin glycoprotein distributions in the *bus-4* surface coat, the underlying cuticle, intestine and the pharynx (Figures 1 and 2). Since *bus-4* is expressed in the hypodermal seam cells, the pharynx and distal intestine [8], these alterations co-localize with loss of genetic function. We found that twice as much *O*-glycan was released from *bus-4* nematode glycoproteins than the parent strain. As the *Ce* core-I glycans are 85 – 90% of *O*-glycans, the majority of the increase was in this class (Figure 4). The *bus-4* surface coat contained double the abundance of *Ce* core-I mucin glycoprotein with the most dramatic increases seen in the head and tail regions (Figure 2A). This increase was associated with expression of the mucins *let-653* and *osm*-8, which were both increased nearly 40 fold. Both are expressed in the hypodermis and seam cells among other tissues. The abundances of soluble mucin in the *bus-4* intestine measured higher although these differences were subtle. Fucosyl glycoconjugates were more concentrated in the hind gut rectum and anus (Figure 2B) showing that soluble glycoproteins of the intestine are altered in abundance and distribution. In contrast to increased glycoconjugate expression seen in soluble mucins, *Ce* core-I glycoprotein abundances seen at the cuticle surface underlying the surface coat were reduced dramatically (Figure 1). Overall the changes seen in mucin distributions and abundances were dramatic.

The *bus*-4 *O*-glycans were shifted in sub-class distributions. *Ce* core-I charged form abundances were reduced by half but neutral *Ce* core-I, neutral fucosyl, and *Ce* core-II forms were increased in relative proportion. Tandem MS examination of the neutral and acidic *Ce* core-I glycans revealed dramatic shifts in isomer distributions as substitution patterns of the core Gal β1,3GalNAc -Ser/Thr were altered. The most significant change was the loss of GlcA substitutions with more subtle shifts in terminal Glc and Gal substitutions. The *bus-4* mucin contained half of the GlcA substitution as wild-type mucin greatly reducing its charge. *C. elegans* can add GlcA to C4 of GalNAc or C3 of Gal in the Gal β1,3GalNAc-Ser/Thr core. In the *bus-4* deficient background there is loss of GlcA at both sites over all but more so at the GalNAc C4 position. The C4 is more often occupied with a Glc substitution in the *bus-4* pool as well. This centers the changes most significantly at the C4 of the core GalNAc. As all members of Pfam01762 are predicted to act on a HexNAc acceptor it is tempting to speculate about the *BUS-4* activity. However, we do not have sufficient evidence to ascribe an activity at this time. We are currently working to purify *BUS-4* and characterize it in vitro.

*N*-glycans were also altered in the *bus-4* strain. Fucosyl forms were increased by 25% relative abundance in their subclass (Figure S10). Detailed structural investigation revealed no differences in *N*-glycoform structures or isomeric distributions. Therefore the quantity but not quality of these glycans was affected. As previously stated, *bus-4* fucosyl *O*-glycans were also increased. This situation, where general glycoprotein fucosylation is affected, occurs at least in part, through a compensatory response at the expression level upstream of *N*- and *O*-glycosylation. GDP-Man 3,5 dehydratase is over 100 fold increased in the *bus-4* strain as shown in this study (Figure 8). This enzyme catalyzes the first committed step in GDP-Fuc synthesis and this nucleotide sugar is the donor substrate in fucosyl glycan synthesis in both pools studied here. Fucosyl *O*-glycans were also increased in the *bus-2* mutant previously studied [27]. In the *bus-2* strain however GDP-Man 3,5 dehydratase was only increased by 20 fold (our unpublished observations). This situation suggests that the glycan and substrate pools are related through complex regulatory processes with multiple levels of control that can affect glycosylation levels and distributions across major glycosylation pathways. Apparently *Ce* core-I glycan pool sensing can impact fucosylation and, in some instances, such as seen here in the *bus-4* mutants, these changes can affect multiple glycosylation pathways.

The *bus-4* mutant presents a severely altered glycosylation profile in the surface coat, underlying cuticle surface and the intestine. *O*-glycan abundance, subclass distribution and isomer distributions are severely altered (Figures 4–7 and S4–S15) as are expression of the key mucins *let-653* and *osm-8*. Subtle changes in *N*-glycosylation further contribute to changes in surface properties. As stated previously, based on anatomical distribution, structural conservation and chemical properties, the *Ce* core-I *O*-glycans are the *C. elegans* equivalent of mucin *O*-glycans. As a critical component of the surface coat they are important in providing a barrier to infection [38]. Previously we reported that *M. nematophilum* must be ingested in order to infect [39]. After traversing the intestine in wild-type nematodes the bacteria will adhere to the rectum and anus forming a plug which expands over the surrounding cuticle. In the altered glycosylation phenotype of the *bus-4* mutant this adherence does not occur. The increased mucin abundance, surface differences seen via live lectin staining, decrease in GlcA-containing glycans, and overall altered *Ce* core-I *O*-glycan structure suggests that the environment at the surface coat is dramatically altered in mucin content. As mucin forms a protective layer against infection this change is likely to be significant. Induction of the mucin *mul-1*has been shown to decrease mortality of *C. elegans* after exposure to *Pseudomonas aeruginosa* [40]. Therefore there is precedence for mucin protection against gram negative bacteria in *C. elegans*. In the *bus-4* strain a higher abundance of secreted mucin is present with only half the normal charge and altered isomer distributions. The cuticle surface was diminished in surface bound mucin glycoproteins and had an altered presentation of other glycoconjugates as shown in fixed-worm lectin staining (Figure 1). *O*-glycosylation changes exist in the intestine as well when *bus-4* expression in the hind gut is lost [8] and altered secreted mucin was also present based on our structural studies (Figures 4 and Table 2) and microscopic studies (Figure 2A). Additionally fucosyl mucin is increased in the rectum and anal regions under some conditions (Figure 2B). *M. nematophilum* does not form a successful infection under this altered glycosylation status.

In higher eukaryotes both commensal and pathogenic bacteria interact with an intestinal environment that appears to form glycoprotein gradients both through the secreted mucin and in underlying tissues [41]. In humans, organisms such as *Pseudomonas aeruginosa* [42] and *E. coli* [43] have developed strategies that target oligosaccharides of the epithelial surfaces. Mucin glycan composition has been shown to vary considerably along the gastrointestinal tract [41] and these glycans change during inflammation [44]. Differences in blood type, which conveys differences in *O*-glycosylation, can alter the microbiome of the human intestine and alter susceptibility to pathogens [45], [46]. These examples demonstrate the importance of glycosylation patterning in the gut for microbial interactions. In nematodes a similar interaction likely exists at the surface coat [38], [47] and intestine [48]. In this regard, the *bus-4* intestine, cuticle surface coat, and underlying cuticle may not be capable of providing the normal cues required for *M. nematophilum* to infect the anus, rectum and associated cuticle. Likewise, the altered properties of the *bus-4* cuticle surface and surface coat are not compatible with adherence of *Y. pestis* and *Y. peudotubercuosis* biofilm. In the case of *Leucobacter* Verde-1 and Verde-2, susceptibilities are flipped. While wildtype nematodes suffer lethal infection by Verde-2 and are resistant to Verde-1, the *bus*-4 mutants are resistant to Verde-2 but suffer lethal infection by Verde-1 [12]. The *bus-4* mutants are also affected in normal interactions, such as mating. All of these changes demonstrate the importance of *bus-4* mediated modifications the absence of which leads to shifts in mucin properties at cell surfaces and secreted mucins of the surface coat.

## Conclusions

To date we have reported alterations in the glycomes of *srf-3*, *bus-2* and *bus-4* mutants. In all cases the *Ce* core-I *O*-glycans were dramatically altered and pathogen resistance is present. Clearly these glycans are required for pathogenic processes of *M.nematophilum*, *Y. pestis* and *Y. peudotubercuosis*. Here we have reported dramatic changes in the glycosylation profile of the surface coat, cuticle surface and intestine in the *bus-4* deficient background. Further examination of *bus-2*, normally expressed in the hind gut and seam cells, also reveals a dramatic increase in mucin *O*-glycan abundance (our unpublished observations) and changes in intestinal glycosylation [13]. These mutants will be useful for further investigation into the function of glycosylation in the surface coat, cuticle and intestine.

## Supporting Information

Figure S1 **ABA staining of acetone fixed N2 and** ***bus-4*** **nematodes.** The images were collected using FITC conjugated ABA and for a shorter time exposure than those in Figure 1. *Ce* core-I *O*-glycans are in the tail region of the cuticle leading up to the anus. The loss of these glycans at the cuticle surface are most dramatic in this region.
(TIF)

Figure S2 **ABA lectin binds to N2 and** ***bus-4 Ce*** **core-I** ***O*** **-glycans.** Glycopeptides from N2 and bus-4 strains were applied to ABA lectin columns, washed with 10 mM phosphate buffer and adsorbed forms eluted using, 100 mM β-methyl galactoside. An aliquot of the samples applied to the column, the washes, and the eluate were subjected to β-elimination, permethylated and analyzed by MALDI-TOF MS. A) N2 sample, B) N2 phosphate buffer wash, C) N2 100 mM β-methyl galactoside eluted glycoforms, D) *bus-4* sample, E) *bus-4* phosphate buffer wash, F) *bus-4* 100 mM β-methyl galactoside eluted glycoforms.
(TIF)

Figure S3 **MALDI-TOF MS analysis of N2 and** ***bus-4*** **permethylated** ***O*** **-glycans.** The glycans of N2 are shown in A and those of *bus-4* in B. Panels on the left include *Ce* core-I neutral, *Ce* core-I charged, and neutral fucosyl forms. Panels to the right are amplified 20 times and contain the *Ce* core-II *O*-glycans. The *bus-4 Ce* core-I charged forms are greatly diminished but *Ce* core-II forms are increased. Key glycans are labeled. See Table 1 for a complete list and Figure 4A and B for relative abundances of *O*-glycans detected in this study.
(TIF)

Figure S4 **The CID MS^3^ analysis of matched N2 and** ***bus-4*** **permethylated** ***m/z*** **502.3 daughter ions of Hex_2_HexNAc_1_-ol,** ***m/z*** **738.6 [M+Na]^+^.** Data were collected under identical conditions using a Thermo LTQ-XL ion trap equipped with an Advion Nanomate sample infusion system. The N2 spectrum appears in the top panel and that of *bus-4* in the bottom panel. Derived structure is shown in the top panel. The ion *m/z* positions and differences in ion intensities are consistent with the same configuration but different monosaccharide compositions.
(TIF)

Figure S5 **The CID MS^3^ analysis of matched N2 and** ***bus-4*** **permethylated** ***m/z*** **463.0 daughter ions of Hex_2_HexNAc_1_-ol,** ***m/z*** **738.6 [M+Na]^+^.** Data were collected under identical conditions using a Thermo LTQ-XL ion trap equipped with an Advion Nanomate sample infusion system. The N2 spectrum appears in the top panel and that of *bus-4* in the bottom panel. Derived structure is shown in the top panel. The ion *m/z* positions and differences in ion intensities are consistent with the same configuration but different monosaccharide compositions.
(TIF)

Figure S6 **The CID MS^3^ analysis of matched N2 and** ***bus-4*** **permethylated** ***m/z*** **706.4 daughter ions of Hex_3_HexNAc_1_-ol,** ***m/z*** **942.6 [M+Na]^+^.** Data were collected under identical conditions using a Thermo LTQ-XL ion trap equipped with an Advion Nanomate sample infusion system. The N2 spectrum appears in the top panel and that of *bus-4* in the bottom panel. The derived structure is shown in the top panel. The ion nearly identical *m/z* positions and ion intensities are consistent with the same configuration for both sources.
(TIF)

Figure S7 **The CID MS^3^ analysis of matched N2 and** ***bus-4*** **permethylated** ***m/z*** **502.3 daughter ions of Hex_3_HexNAc_1_-ol,** ***m/z*** **942.6 [M+Na]^+^.** Data were collected under identical conditions using a Thermo LTQ-XL ion trap equipped with an Advion Nanomate sample infusion system. The N2 spectrum appears in the top panel and that of *bus-4* in the bottom panel. Derived structure is shown in the top panel. The ion abundances and nearly identical *m/z* positions and intensities are consistent with the same configuration for both sources.
(TIF)

Figure S8 **The CID MS^3^ analysis of matched N2 and** ***bus-4*** **permethylated** ***m/z*** **463.0 daughter ions of Hex_3_HexNAc_1_-ol,** ***m/z*** **942.6 [M+Na]^+^.** Data were collected under identical conditions using a Thermo LTQ-XL ion trap equipped with an Advion Nanomate sample infusion system. The N2 spectrum appears in the top panel and that of *bus-4* in the bottom panel. The derived structures are shown in the top panel. The ion abundances and nearly identical *m/z* positions and intensities are consistent with the same configuration for both sources.
(TIF)

Figure S9 **The CID MS^2^ analysis of matched N2 and** ***bus-4*** **permethylated** ***m/z*** **Hex_4_HexNAc_1_-ol,** ***m/z*** **1146.6 [M+Na]^+^.** Data were collected under identical conditions using a Thermo LTQ-XL ion trap equipped with an Advion Nanomate sample infusion system. The N2 spectrum appears in the top panel and that of *bus-4* in the bottom panel. The derived structures are shown in the top panel. The ion abundances and nearly identical *m/z* positions and intensities are consistent with the same configuration for both sources.
(TIF)

Figure S10 **The CID MS^3^ analysis of matched N2 and** ***bus-4*** **permethylated** ***m/z*** **681.4 daughter ions of HexA_1_Hex_3_HexNAc_1_-ol,** ***m/z*** **1160.7[M+Na]^+^.** Data were collected under identical conditions using a Thermo LTQ-XL ion trap equipped with an Advion Nanomate sample infusion system. The N2 spectrum appears in the left panel and that of *bus-4* in the right panel. The derived structure is shown boxed at top center. The nearly identical ion abundances, *m/z* positions and intensities are consistent with the same configuration for both sources.
(TIF)

Figure S11 **The CID MS^3^ analysis of matched N2 and** ***bus-4*** **permethylated** ***m/z*** **502.3 daughter ions of HexA_1_Hex_3_HexNAc_1_-ol,** ***m/z*** **1160.7 [M+Na]^+^.** Data were collected under identical conditions using a Thermo LTQ-XL ion trap equipped with an Advion Nanomate sample infusion system. The N2 spectrum appears in the top panel and that of *bus-4* in the bottom panel. Derived structure is shown in the top panel. The ion *m/z* positions and differences in ion intensities are consistent with the same configuration but different monosaccharide compositions.
(TIF)

Figure S12 **The CID MS^3^ analysis of matched N2 and** ***bus-4*** **permethylated** ***m/z*** **720.4 daughter ions of HexA_1_Hex_3_HexNAc_1_-ol,** ***m/z*** **1160.7 [M+Na]^+^.** Data were collected under identical conditions using a Thermo LTQ-XL ion trap equipped with an Advion Nanomate sample infusion system. The N2 spectrum appears in the top panel and that of *bus-4* in the bottom panel. The derived structure is shown in the top panel. The nearly identical ion *m/z* positions and ion intensities are consistent with the same configuration.
(TIF)

Figure S13 **The CID MS^3^ analysis of matched N2 and** ***bus-4*** **permethylated** ***m/z*** **885.5 daughter ions of HexA_1_Hex_3_HexNAc_1_-ol,** ***m/z*** **1160.7 [M+Na]^+^.** Data were collected under identical conditions using a Thermo LTQ-XL ion trap equipped with an Advion Nanomate sample infusion system. The N2 spectrum appears in the top panel and that of *bus-4* in the bottom panel. The derived structure is shown in the top panel. The nearly identical ion *m/z* positions and ion intensities are consistent with the same configuration.
(TIF)

Figure S14 **The CID MS^2^ analysis of matched N2 and** ***bus-4*** **permethylated** ***m/z*** **HexA_1_Hex_4_HexNAc_1_-ol,** ***m/z*** **1364.8 [M+Na]^+^.** Data were collected under identical conditions using a Thermo LTQ-XL ion trap equipped with an Advion Nanomate sample infusion system. The N2 spectrum appears in the top panel and that of *bus-4* in the bottom panel. The derived structure is shown in the top panel. The ion abundances and nearly identical *m/z* positions and intensities are consistent with the same configuration for both sources.
(TIF)

Figure S15 **The CID MS^3^ analysis of matched N2 and** ***bus-4*** **permethylated** ***m/z*** **502.3 daughter ions of HexA_1_Hex_4_HexNAc_1_-ol,** ***m/z*** **1364 [M+Na]^+^.** Data were collected under identical conditions using a Thermo LTQ-XL ion trap equipped with an Advion Nanomate sample infusion system. The N2 spectrum appears in the top panel and that of *bus-4* in the bottom panel. Derived structure is shown in the top panel. The ion *m/z* positions and differences in ion intensities are consistent with the same configuration but different monosaccharide compositions.
(TIF)

Figure S16 ***N*** **-glycan subclass abundances detected in this study.** Gray bars are N2 derived N-glycan abundances. Black bars are *bus-4* derived N-glycan abundances. Glycans in the top panel were released using PNGase F and the bottom panel were released subsequently using PNGase A.
(TIF)

Figure S17 **GNA staining of acetone fixed N2 and** ***bus-4*** **nematodes.** The images were collected using FITC conjugated GNA. Staining is more intense in the *bus-4* nematodes and most pronounced in the tail.
(TIF)

Figure S18 **UEA-1 staining of acetone fixed N2 and** ***bus-4*** **nematodes.** The images were collected using FITC conjugated GNA. Staining is mildly more intense in the *bus-4* nematodes near the tail region.
(TIF)

Figure S19 **WGA staining of acetone fixed N2 and** ***bus-4*** **nematodes.** The images were collected using FITC conjugated WGA. Little to no difference in staining was observed.
(TIF)

Figure S20 **AAA staining of acetone fixed N2 and** ***bus-4*** **nematodes.** The images were collected using FITC conjugated AAA. Little to no difference in staining was observed.
(TIF)

Figure S21 **MALDI-TOF MS analysis of permethylated** ***bus-4*** **and N2 PNGase F released** ***N*** **-glycans.** Top panel: N2 PNGase F released glycans spectrum; Center panel*bus-4* PNGase F released *N*-glycans; lower panel comparative histogram.
(TIF)

Figure S22 **MALDI-TOF MS analysis of permethylated** ***bus-4*** **and N2 PNGase A released** ***N*** **-glycans.** Top panel: N2 PNGase F released glycans spectrum; Center panel*bus-4* PNGase F released *N*-glycans; lower panel comparative histogram.
(TIF)

Table S1 **^a^Glycans molecular ions that are only observed in PNGaseA releases.** ^b^ All ions are sodium adducts.
(DOC)
