# Supplementary figures and images for: Caenorhabditis elegans Bacterial Pathogen Resistant bus-4 Mutants Produce Altered Mucins
Source: PLoS One. 2014 Oct 8;9(10):e107250. doi: 10.1371/journal.pone.0107250 (PMC4189790; doi:10.1371/journal.pone.0107250)

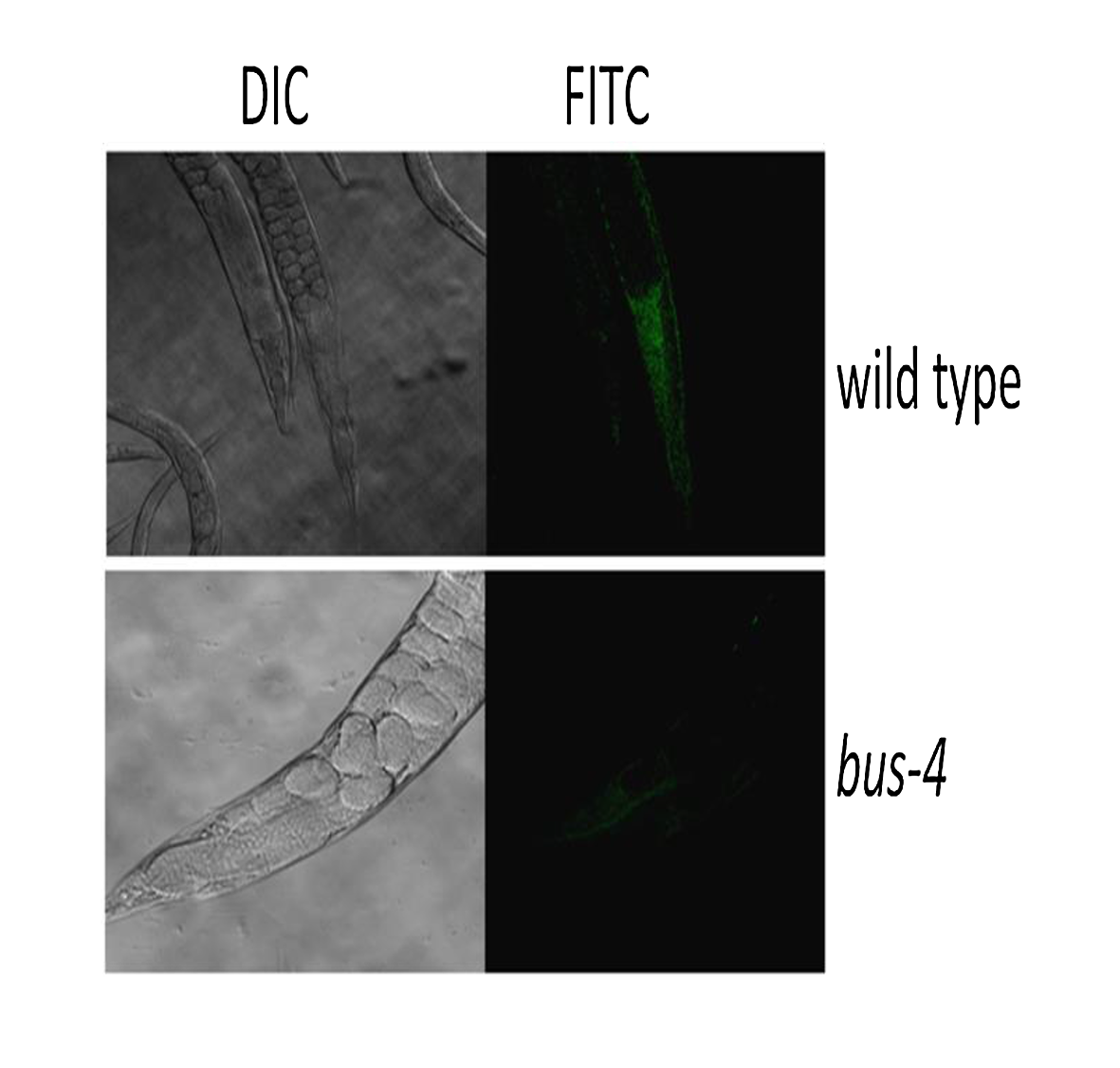

Supplement: Figure S1 — ABA staining of acetone fixed N2 and bus-4 nematodes. The images were collected using FITC conjugated ABA and for a shorter time exposure than those in Figure 1. Ce core-I O-glycans are in the tail region of the cuticle leading up to the anus. The loss of these glycans at the cuticle surface are most dramatic in this region. (TIF) [file pone.0107250.s001.tif]

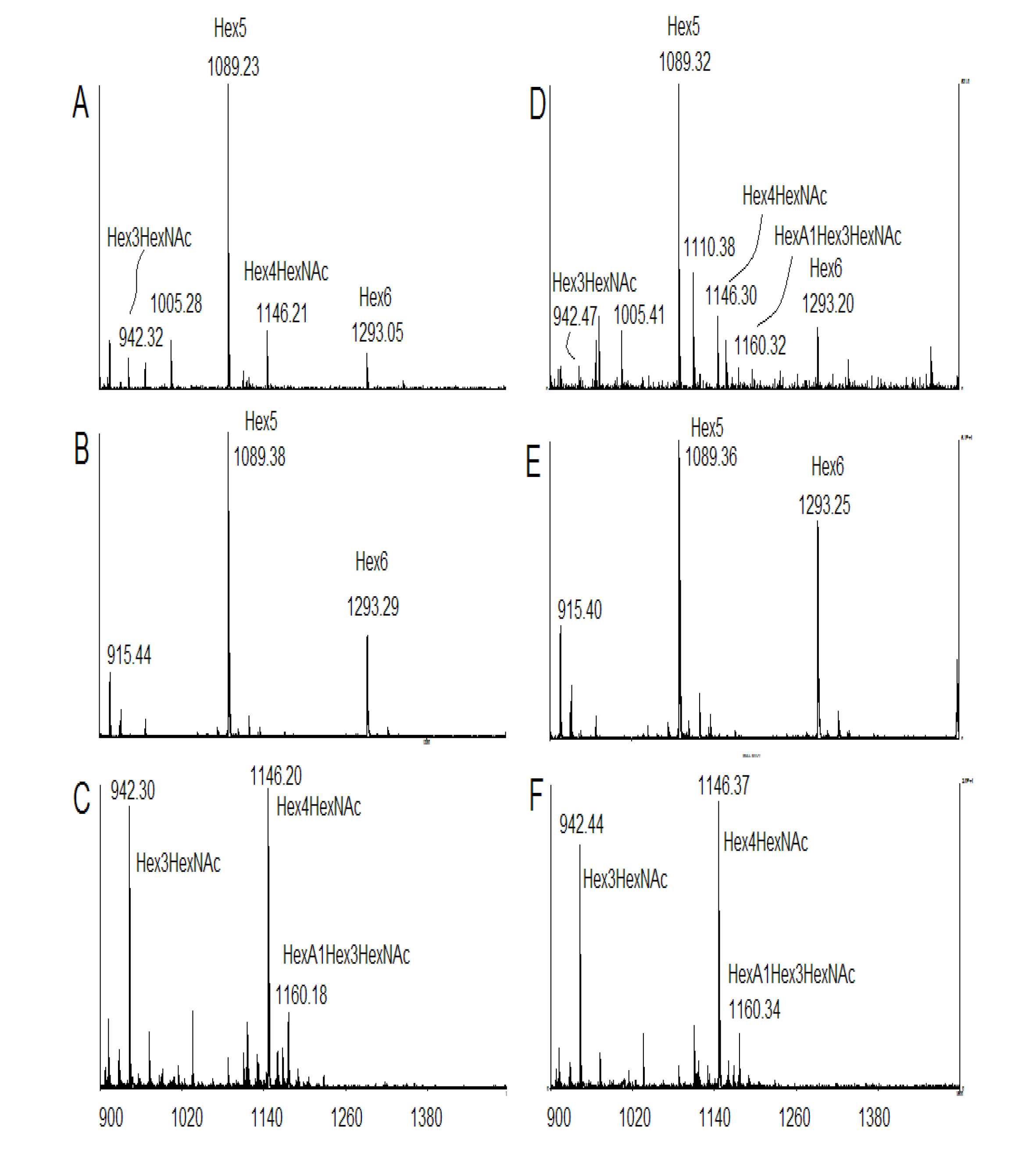

Supplement: Figure S2 — ABA lectin binds to N2 and bus-4 Ce core-I O -glycans. Glycopeptides from N2 and bus-4 strains were applied to ABA lectin columns, washed with 10 mM phosphate buffer and adsorbed forms eluted using, 100 mM β-methyl galactoside. An aliquot of the samples applied to the column, the washes, and the eluate were subjected to β-elimination, permethylated and analyzed by MALDI-TOF MS. A) N2 sample, B) N2 phosphate buffer wash, C) N2 100 mM β-methyl galactoside eluted glycoforms, D) bus-4 sample, E) bus-4 phosphate buffer wash, F) bus-4 100 mM β-methyl galactoside eluted glycoforms. (TIF) [file pone.0107250.s002.tif]

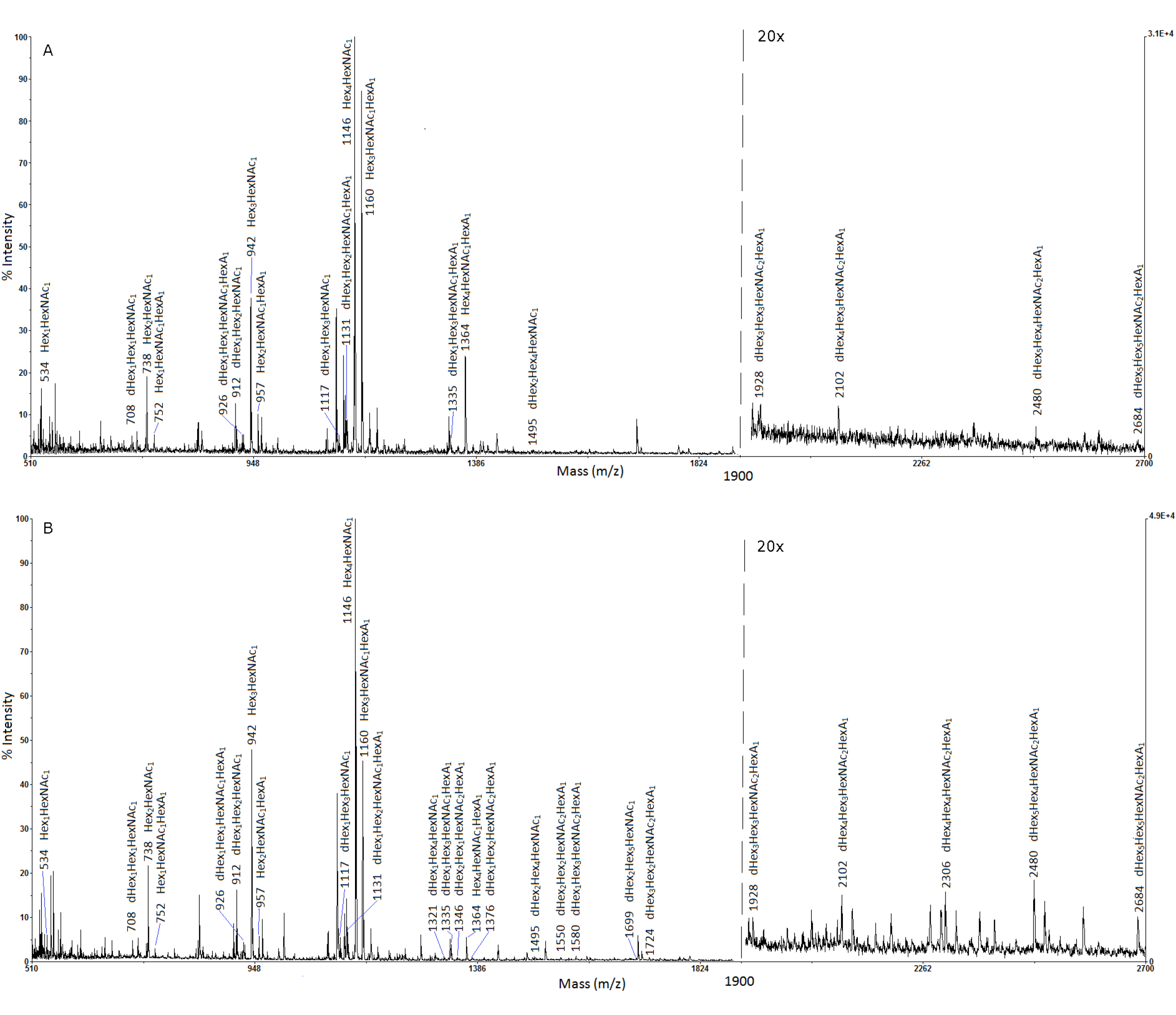

Supplement: Figure S3 — MALDI-TOF MS analysis of N2 and bus-4 permethylated O -glycans. The glycans of N2 are shown in A and those of bus-4 in B. Panels on the left include Ce core-I neutral, Ce core-I charged, and neutral fucosyl forms. Panels to the right are amplified 20 times and contain the Ce core-II O-glycans. The bus-4 Ce core-I charged forms are greatly diminished but Ce core-II forms are increased. Key glycans are labeled. See Table 1 for a complete list and Figure 4A and B for relative abundances of O-glycans detected in this study. (TIF) [file pone.0107250.s003.tif]

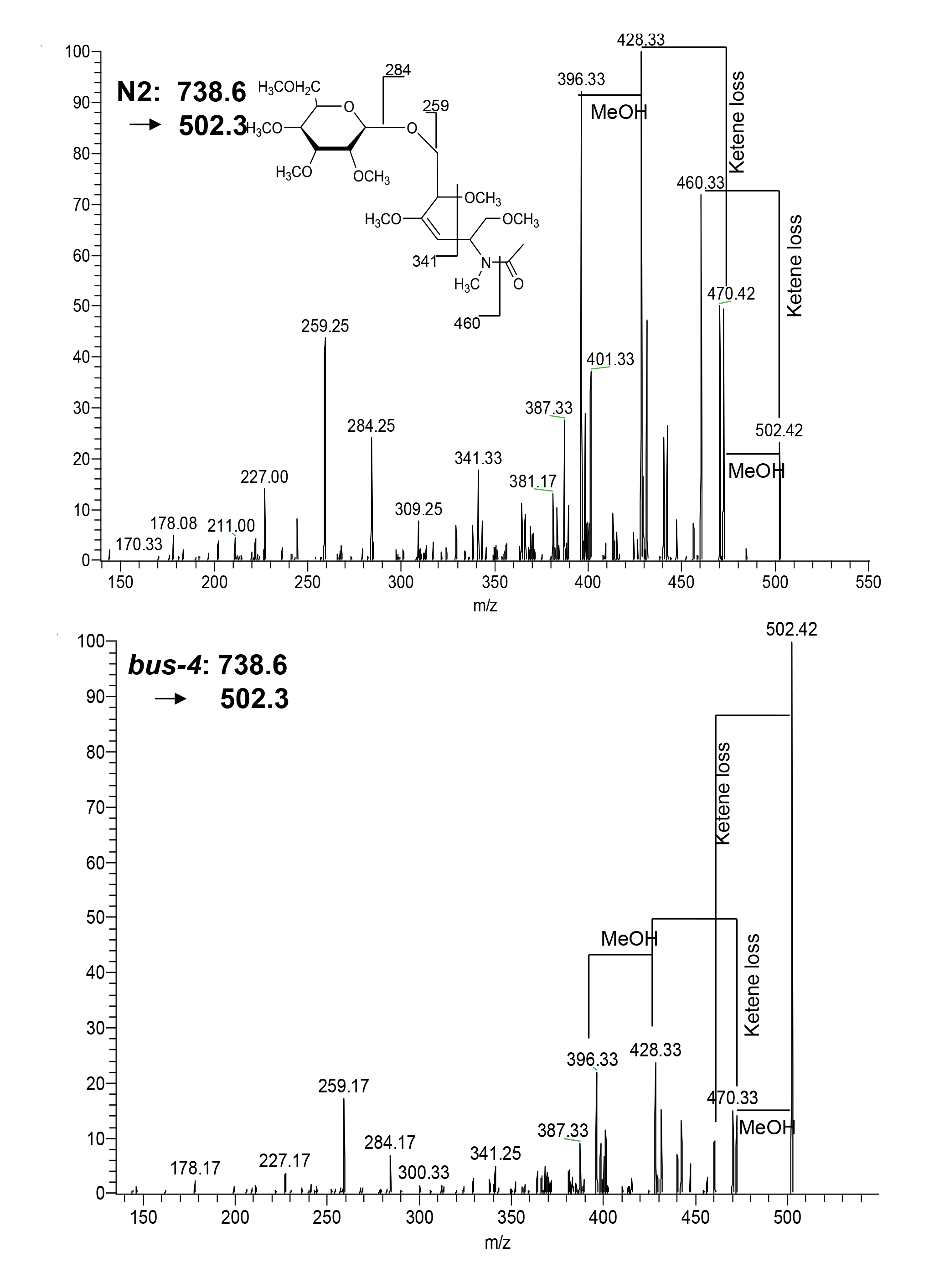

Supplement: Figure S4 — The CID MS3 analysis of matched N2 and bus-4 permethylated m/z 502.3 daughter ions of Hex2HexNAc1-ol, m/z 738.6 [M+Na]+. Data were collected under identical conditions using a Thermo LTQ-XL ion trap equipped with an Advion Nanomate sample infusion system. The N2 spectrum appears in the top panel and that of bus-4 in the bottom panel. Derived structure is shown in the top panel. The ion m/z positions and differences in ion intensities are consistent with the same configuration but different monosaccharide compositions. (TIF) [file pone.0107250.s004.tif]

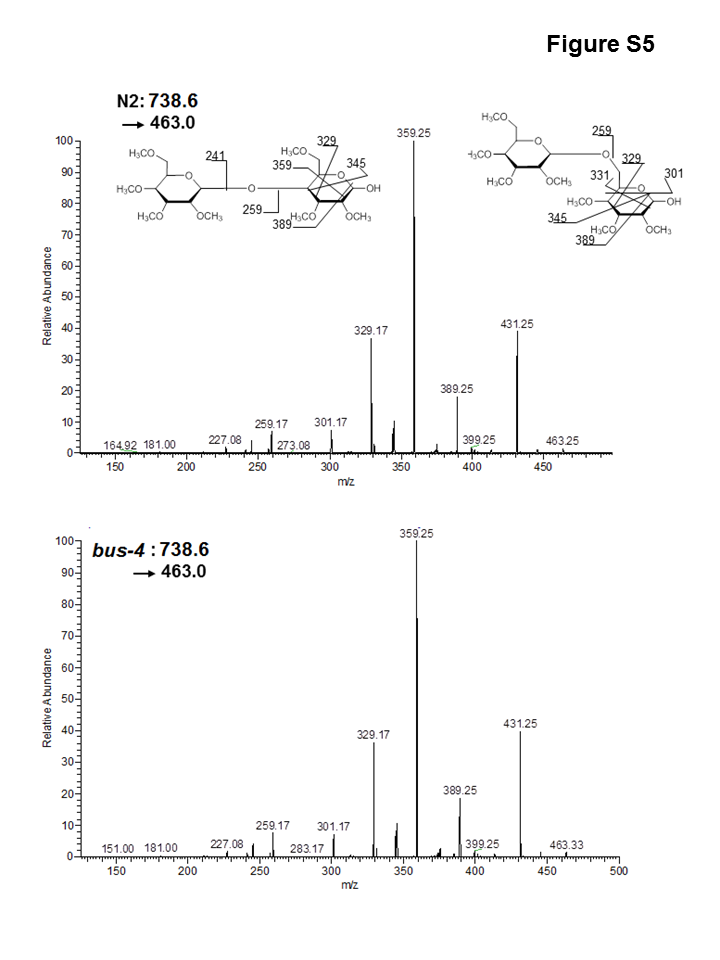

Supplement: Figure S5 — The CID MS3 analysis of matched N2 and bus-4 permethylated m/z 463.0 daughter ions of Hex2HexNAc1-ol, m/z 738.6 [M+Na]+. Data were collected under identical conditions using a Thermo LTQ-XL ion trap equipped with an Advion Nanomate sample infusion system. The N2 spectrum appears in the top panel and that of bus-4 in the bottom panel. Derived structure is shown in the top panel. The ion m/z positions and differences in ion intensities are consistent with the same configuration but different monosaccharide compositions. (TIF) [file pone.0107250.s005.tif]

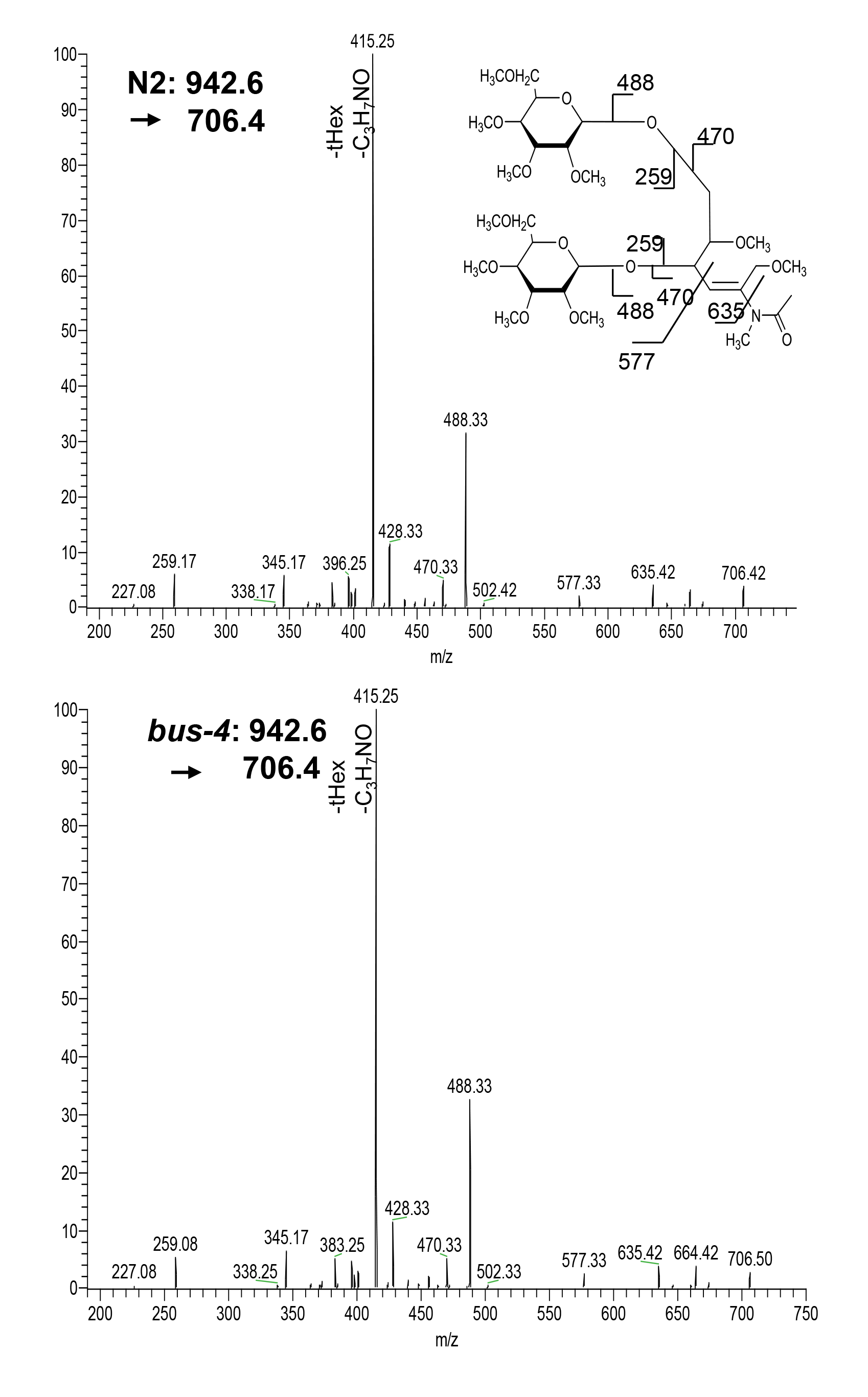

Supplement: Figure S6 — The CID MS3 analysis of matched N2 and bus-4 permethylated m/z 706.4 daughter ions of Hex3HexNAc1-ol, m/z 942.6 [M+Na]+. Data were collected under identical conditions using a Thermo LTQ-XL ion trap equipped with an Advion Nanomate sample infusion system. The N2 spectrum appears in the top panel and that of bus-4 in the bottom panel. The derived structure is shown in the top panel. The ion nearly identical m/z positions and ion intensities are consistent with the same configuration for both sources. (TIF) [file pone.0107250.s006.tif]

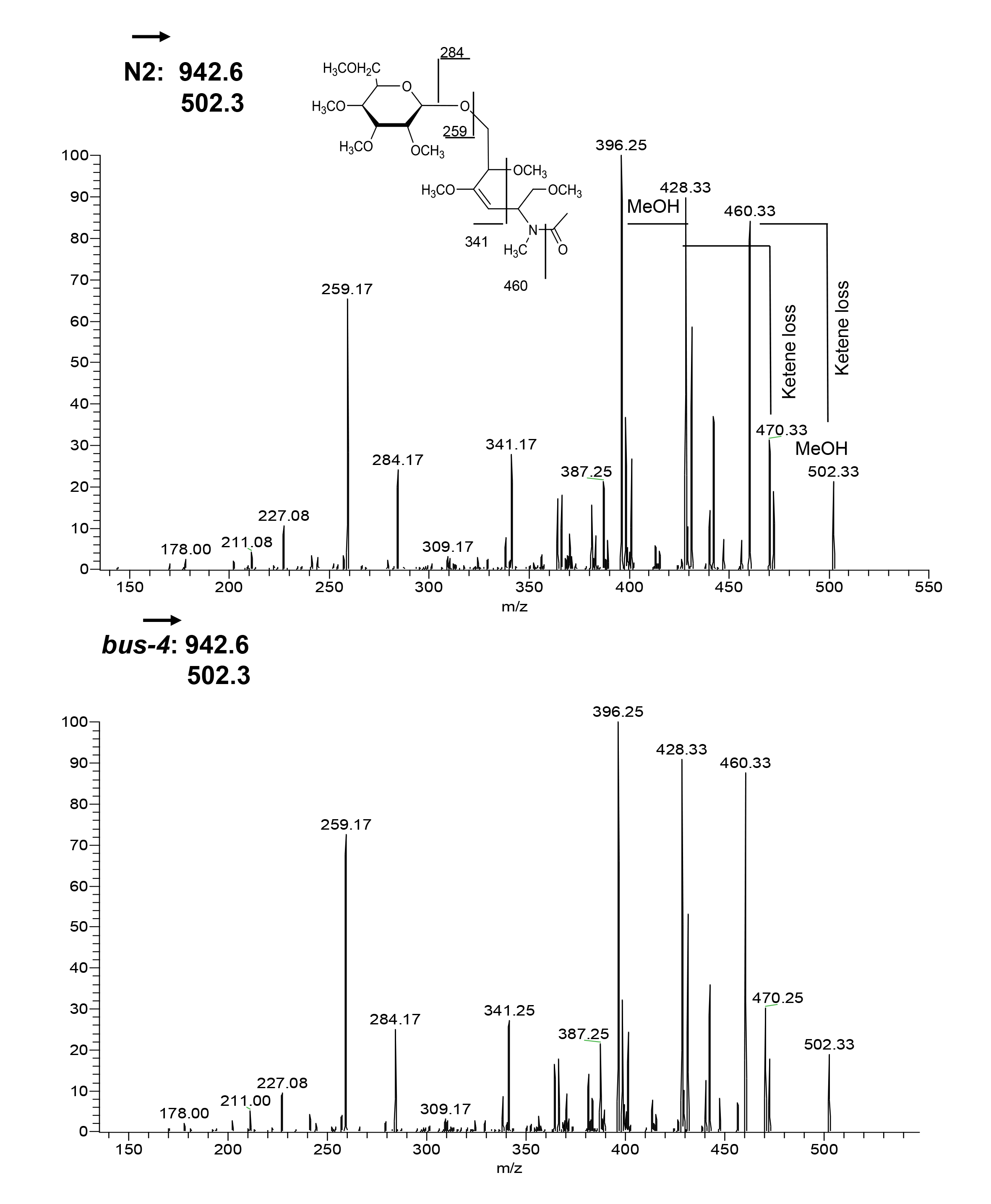

Supplement: Figure S7 — The CID MS3 analysis of matched N2 and bus-4 permethylated m/z 502.3 daughter ions of Hex3HexNAc1-ol, m/z 942.6 [M+Na]+. Data were collected under identical conditions using a Thermo LTQ-XL ion trap equipped with an Advion Nanomate sample infusion system. The N2 spectrum appears in the top panel and that of bus-4 in the bottom panel. Derived structure is shown in the top panel. The ion abundances and nearly identical m/z positions and intensities are consistent with the same configuration for both sources. (TIF) [file pone.0107250.s007.tif]

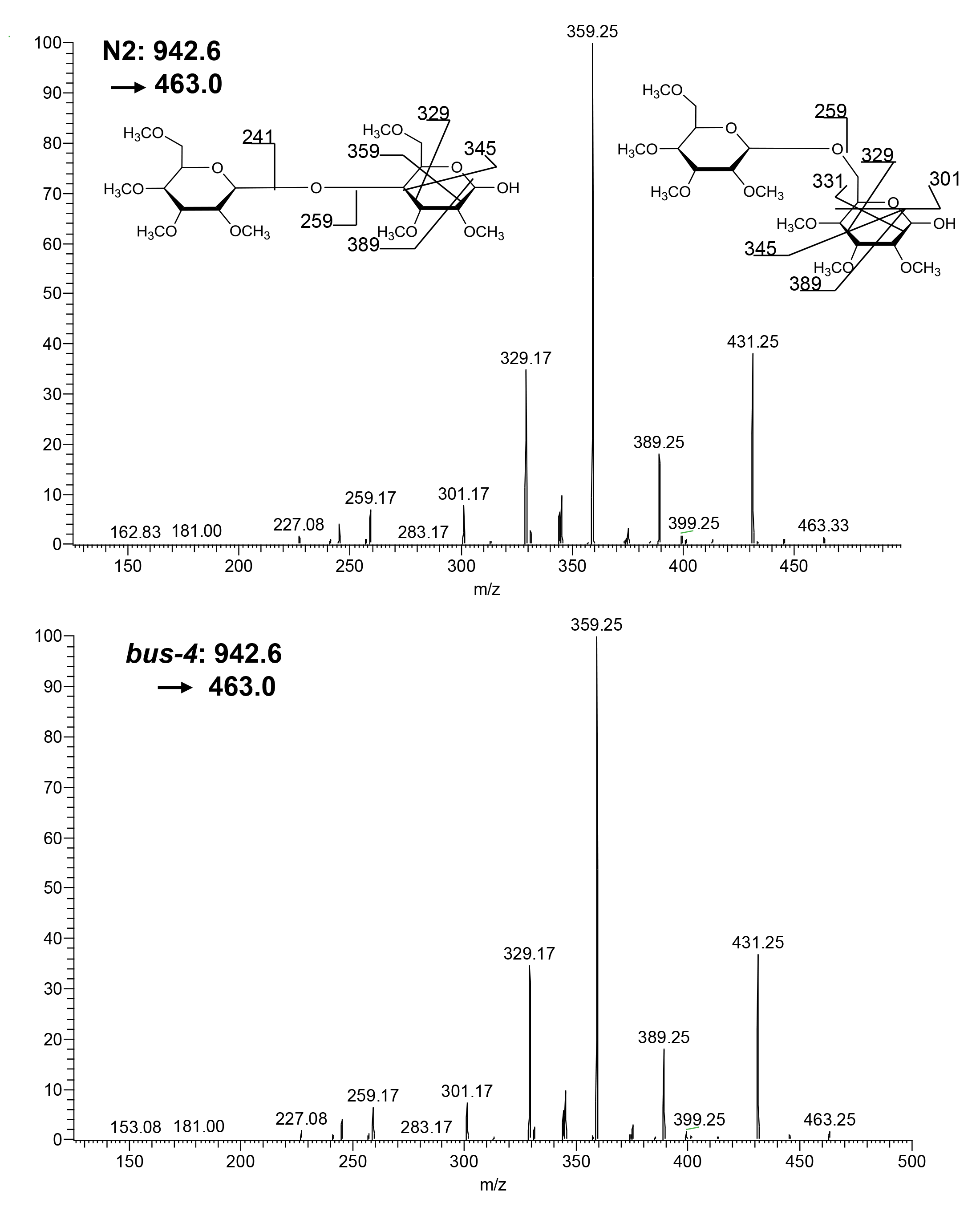

Supplement: Figure S8 — The CID MS3 analysis of matched N2 and bus-4 permethylated m/z 463.0 daughter ions of Hex3HexNAc1-ol, m/z 942.6 [M+Na]+. Data were collected under identical conditions using a Thermo LTQ-XL ion trap equipped with an Advion Nanomate sample infusion system. The N2 spectrum appears in the top panel and that of bus-4 in the bottom panel. The derived structures are shown in the top panel. The ion abundances and nearly identical m/z positions and intensities are consistent with the same configuration for both sources. (TIF) [file pone.0107250.s008.tif]

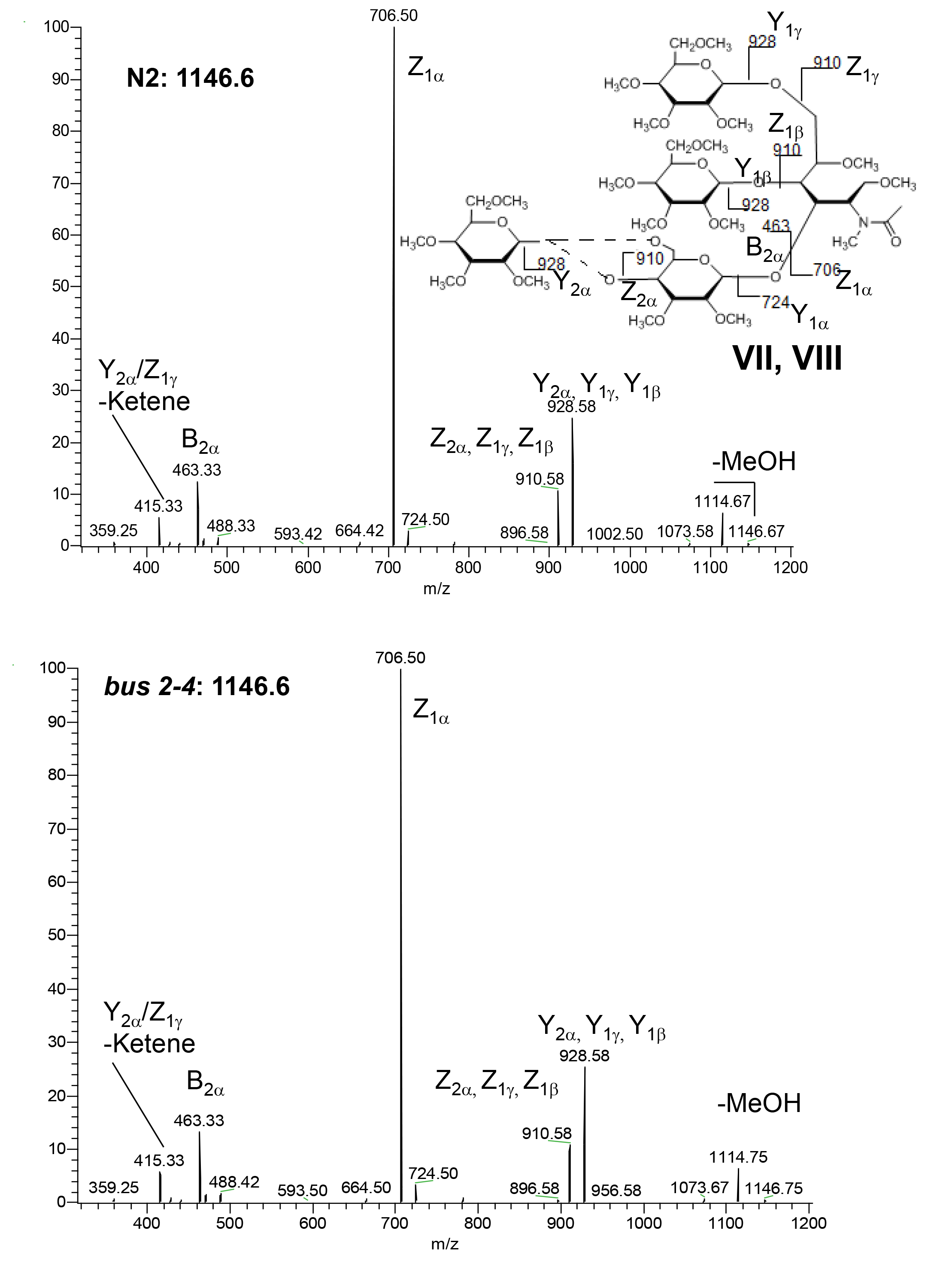

Supplement: Figure S9 — The CID MS2 analysis of matched N2 and bus-4 permethylated m/z Hex4HexNAc1-ol, m/z 1146.6 [M+Na]+. Data were collected under identical conditions using a Thermo LTQ-XL ion trap equipped with an Advion Nanomate sample infusion system. The N2 spectrum appears in the top panel and that of bus-4 in the bottom panel. The derived structures are shown in the top panel. The ion abundances and nearly identical m/z positions and intensities are consistent with the same configuration for both sources. (TIF) [file pone.0107250.s009.tif]

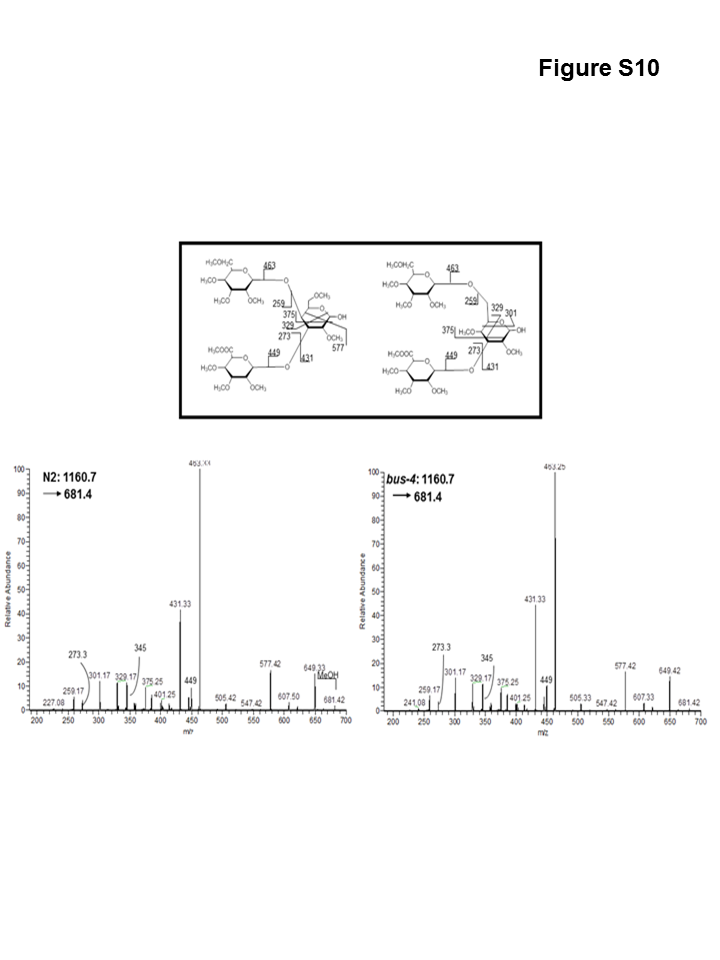

Supplement: Figure S10 — The CID MS3 analysis of matched N2 and bus-4 permethylated m/z 681.4 daughter ions of HexA1Hex3HexNAc1-ol, m/z 1160.7[M+Na]+. Data were collected under identical conditions using a Thermo LTQ-XL ion trap equipped with an Advion Nanomate sample infusion system. The N2 spectrum appears in the left panel and that of bus-4 in the right panel. The derived structure is shown boxed at top center. The nearly identical ion abundances, m/z positions and intensities are consistent with the same configuration for both sources. (TIF) [file pone.0107250.s010.tif]

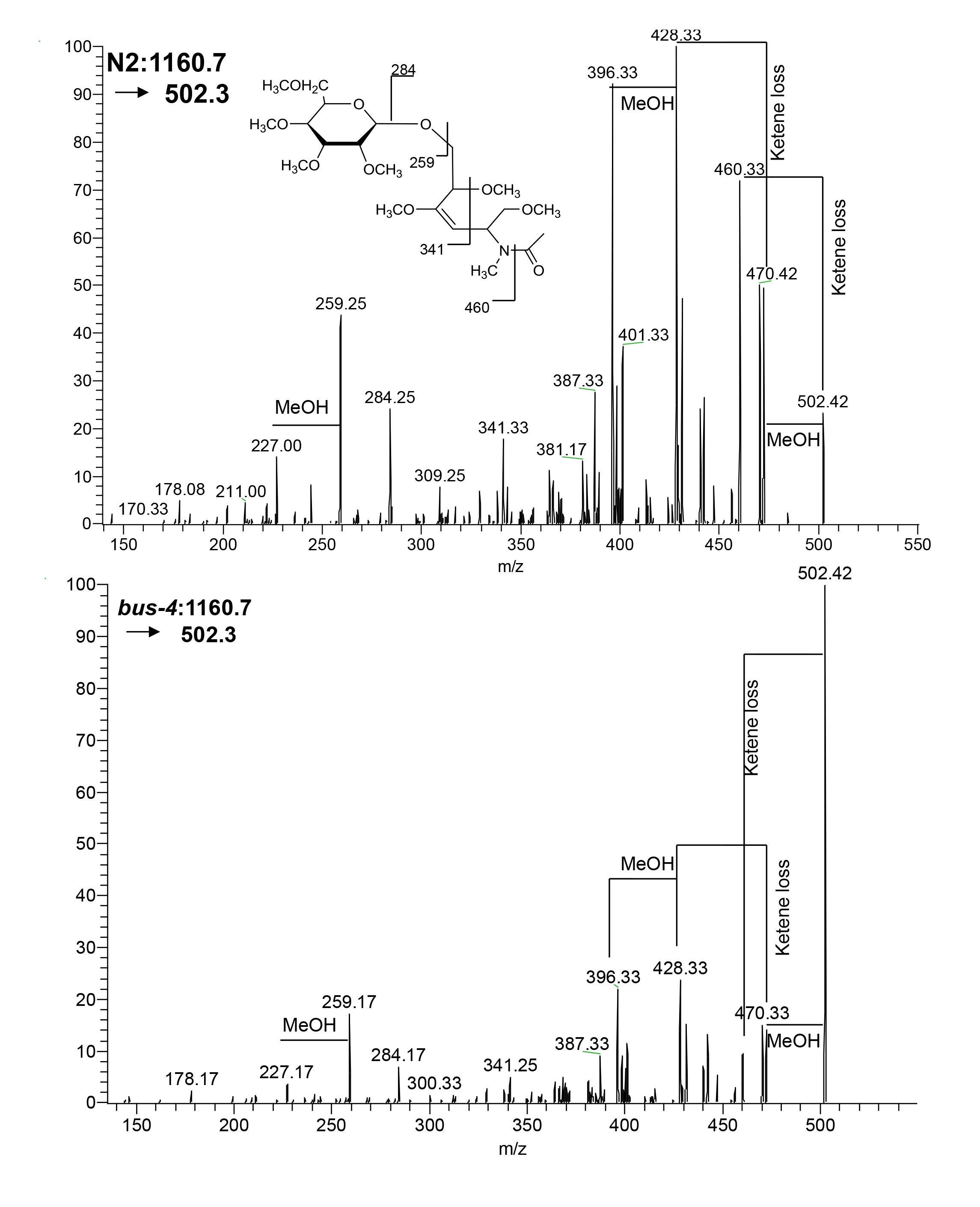

Supplement: Figure S11 — The CID MS3 analysis of matched N2 and bus-4 permethylated m/z 502.3 daughter ions of HexA1Hex3HexNAc1-ol, m/z 1160.7 [M+Na]+. Data were collected under identical conditions using a Thermo LTQ-XL ion trap equipped with an Advion Nanomate sample infusion system. The N2 spectrum appears in the top panel and that of bus-4 in the bottom panel. Derived structure is shown in the top panel. The ion m/z positions and differences in ion intensities are consistent with the same configuration but different monosaccharide compositions. (TIF) [file pone.0107250.s011.tif]

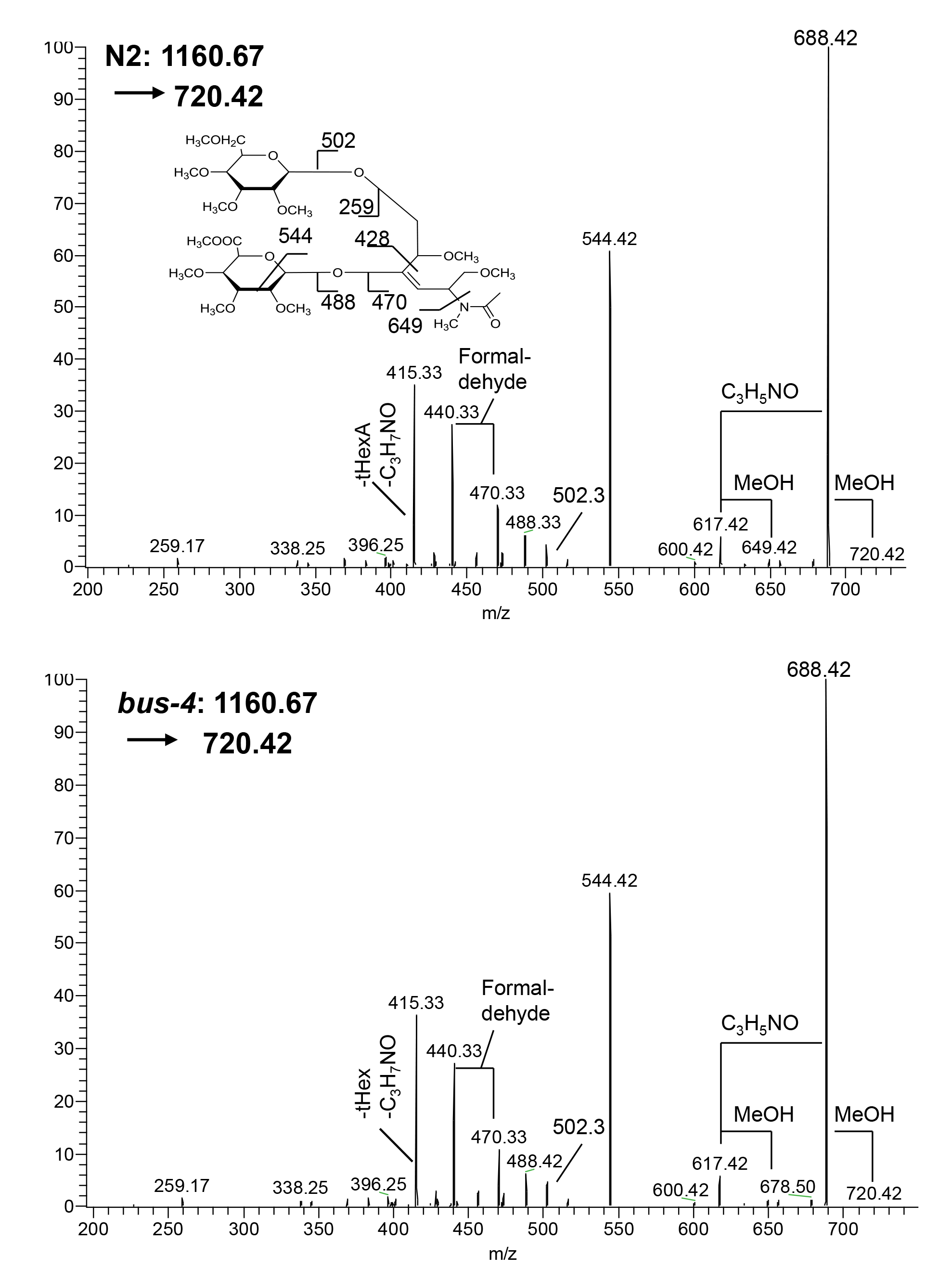

Supplement: Figure S12 — The CID MS3 analysis of matched N2 and bus-4 permethylated m/z 720.4 daughter ions of HexA1Hex3HexNAc1-ol, m/z 1160.7 [M+Na]+. Data were collected under identical conditions using a Thermo LTQ-XL ion trap equipped with an Advion Nanomate sample infusion system. The N2 spectrum appears in the top panel and that of bus-4 in the bottom panel. The derived structure is shown in the top panel. The nearly identical ion m/z positions and ion intensities are consistent with the same configuration. (TIF) [file pone.0107250.s012.tif]

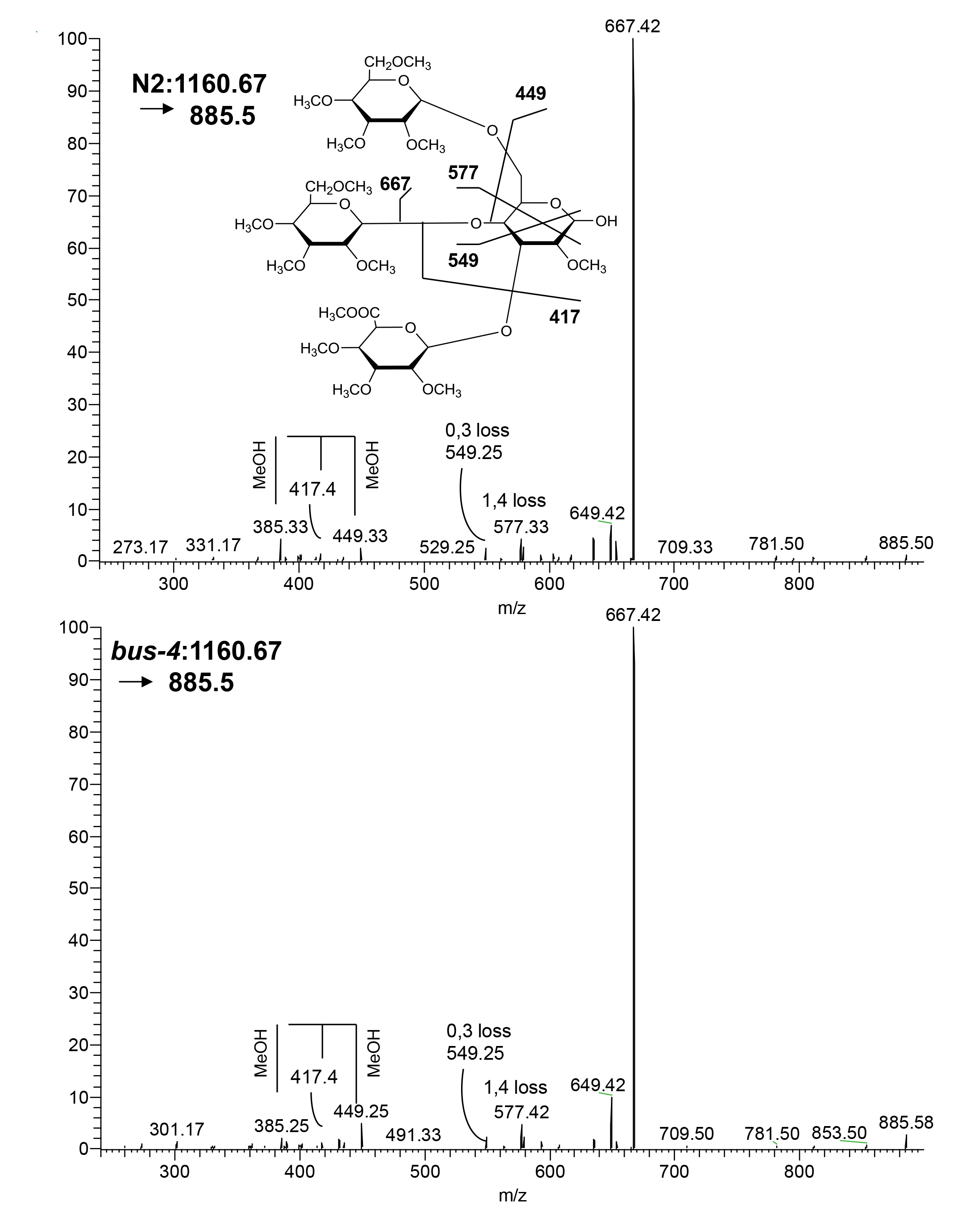

Supplement: Figure S13 — The CID MS3 analysis of matched N2 and bus-4 permethylated m/z 885.5 daughter ions of HexA1Hex3HexNAc1-ol, m/z 1160.7 [M+Na]+. Data were collected under identical conditions using a Thermo LTQ-XL ion trap equipped with an Advion Nanomate sample infusion system. The N2 spectrum appears in the top panel and that of bus-4 in the bottom panel. The derived structure is shown in the top panel. The nearly identical ion m/z positions and ion intensities are consistent with the same configuration. (TIF) [file pone.0107250.s013.tif]

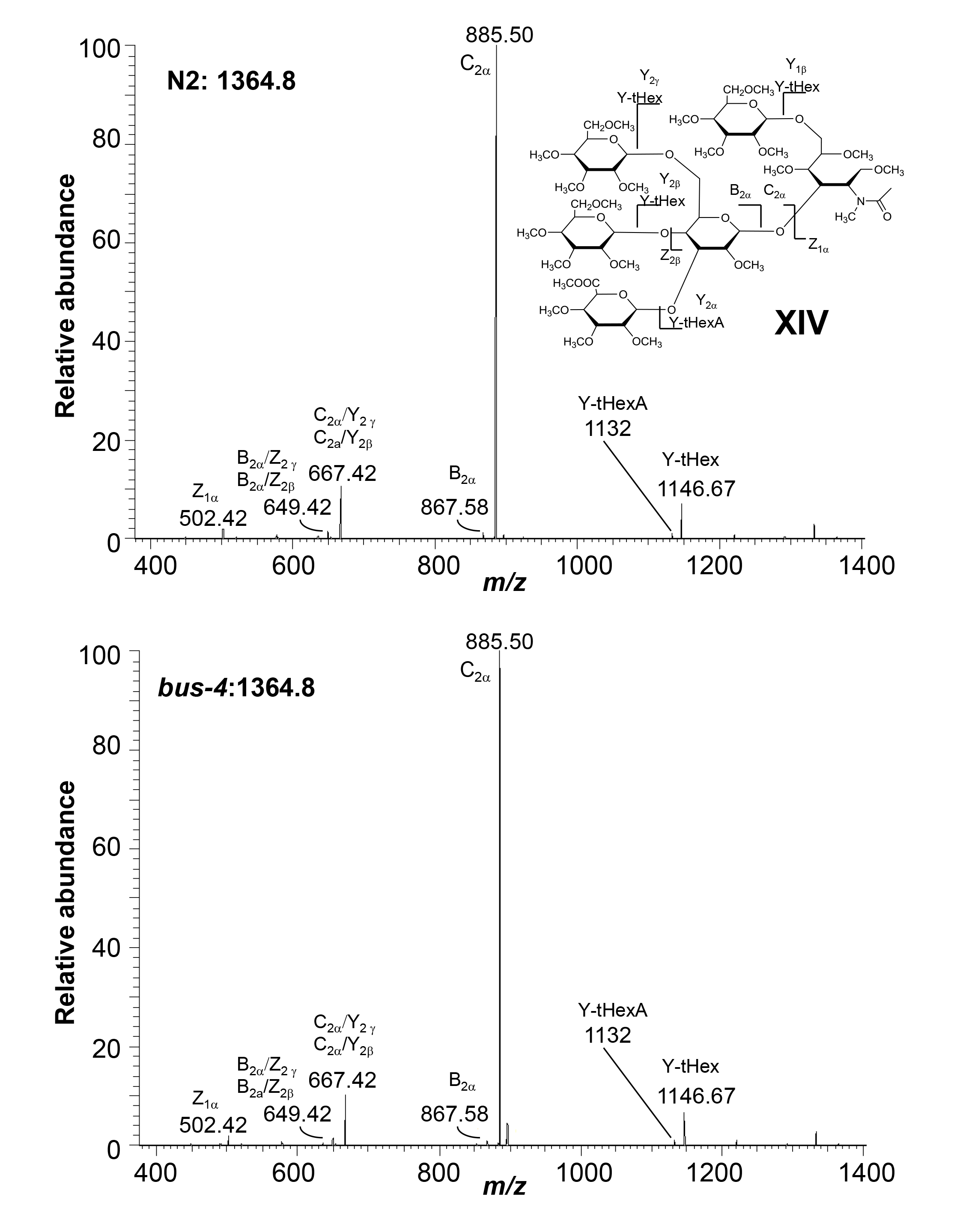

Supplement: Figure S14 — The CID MS2 analysis of matched N2 and bus-4 permethylated m/z HexA1Hex4HexNAc1-ol, m/z 1364.8 [M+Na]+. Data were collected under identical conditions using a Thermo LTQ-XL ion trap equipped with an Advion Nanomate sample infusion system. The N2 spectrum appears in the top panel and that of bus-4 in the bottom panel. The derived structure is shown in the top panel. The ion abundances and nearly identical m/z positions and intensities are consistent with the same configuration for both sources. (TIF) [file pone.0107250.s014.tif]

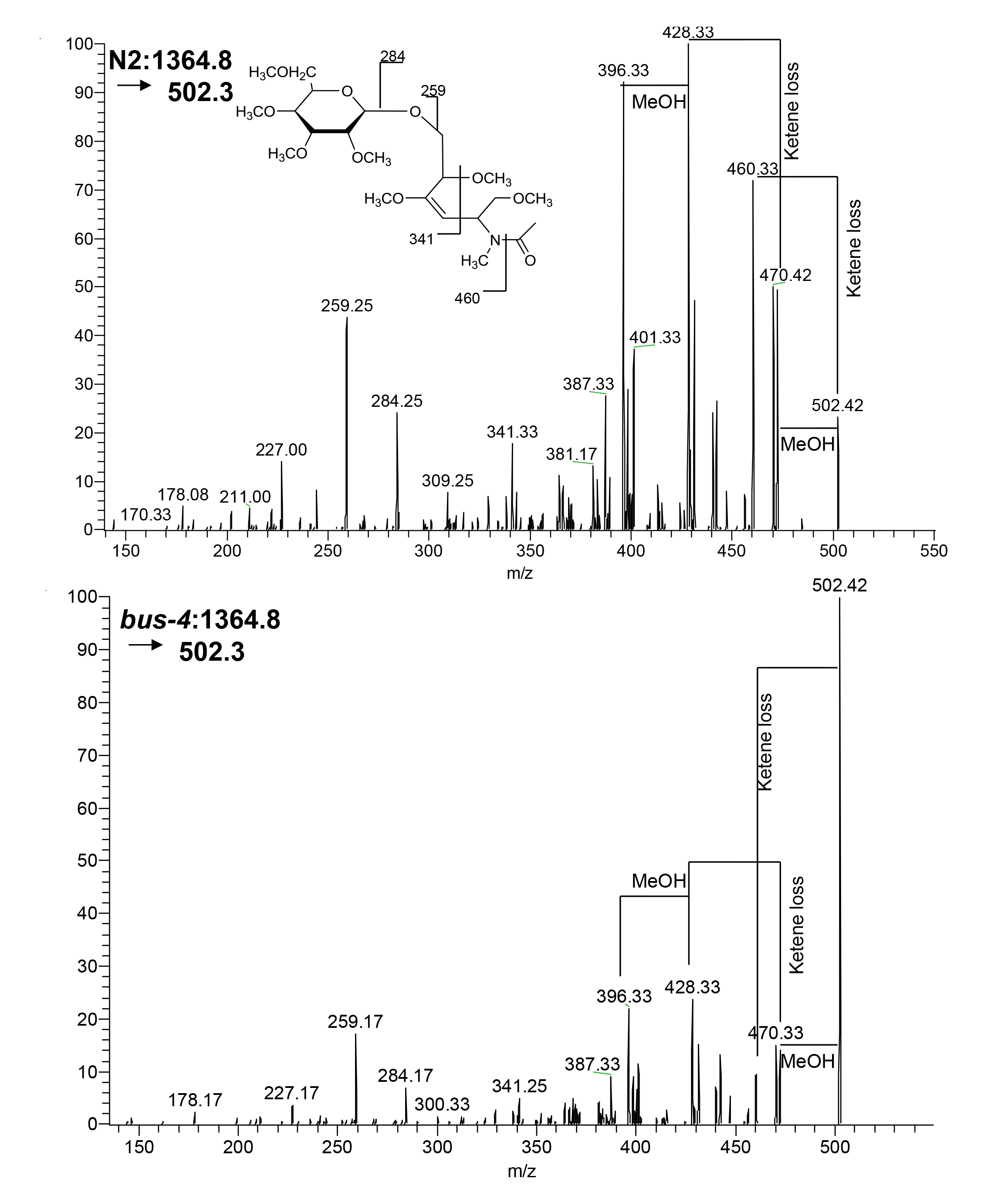

Supplement: Figure S15 — The CID MS3 analysis of matched N2 and bus-4 permethylated m/z 502.3 daughter ions of HexA1Hex4HexNAc1-ol, m/z 1364 [M+Na]+. Data were collected under identical conditions using a Thermo LTQ-XL ion trap equipped with an Advion Nanomate sample infusion system. The N2 spectrum appears in the top panel and that of bus-4 in the bottom panel. Derived structure is shown in the top panel. The ion m/z positions and differences in ion intensities are consistent with the same configuration but different monosaccharide compositions. (TIF) [file pone.0107250.s015.tif]

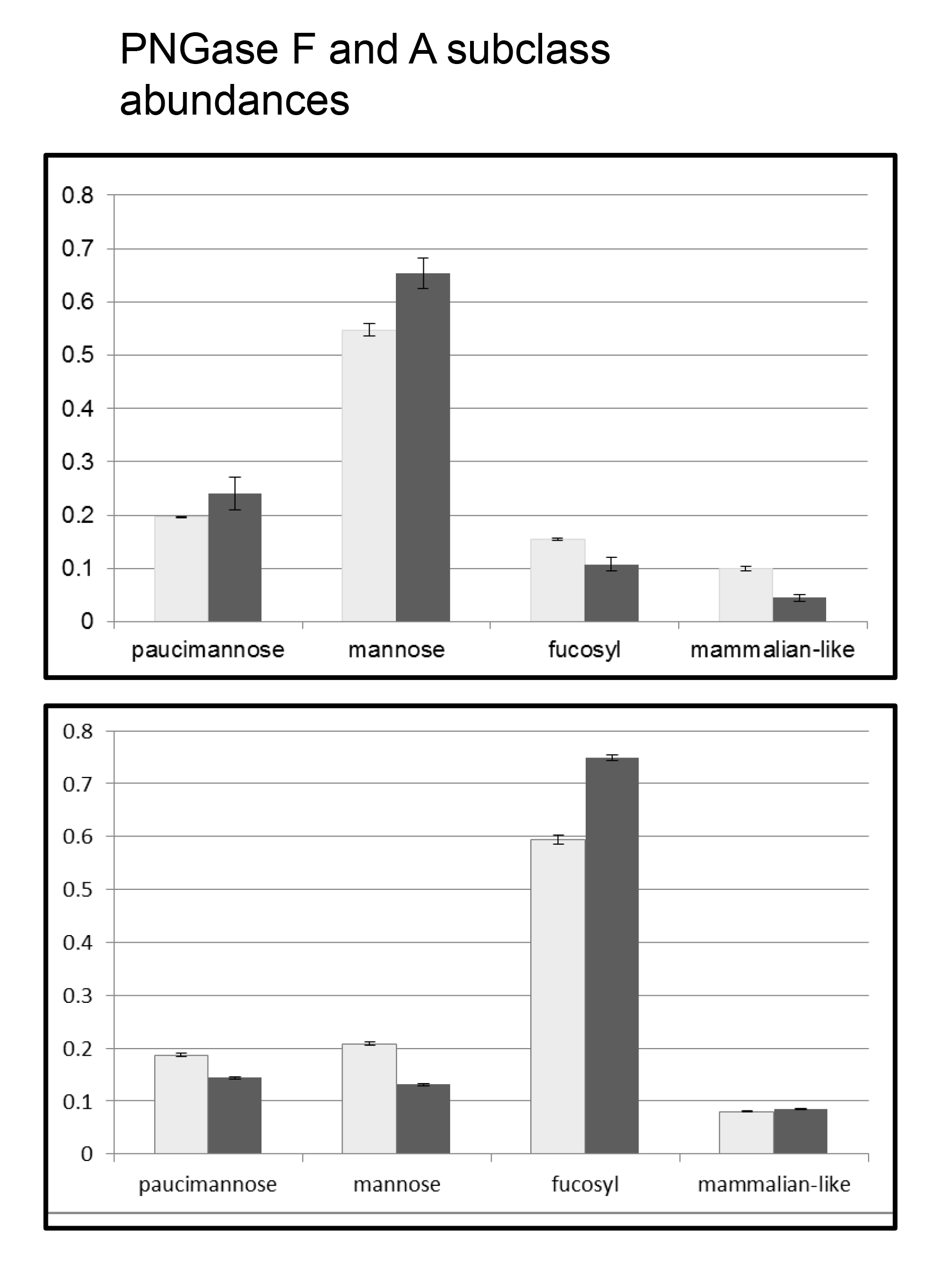

Supplement: Figure S16 — N -glycan subclass abundances detected in this study. Gray bars are N2 derived N-glycan abundances. Black bars are bus-4 derived N-glycan abundances. Glycans in the top panel were released using PNGase F and the bottom panel were released subsequently using PNGase A. (TIF) [file pone.0107250.s016.tif]

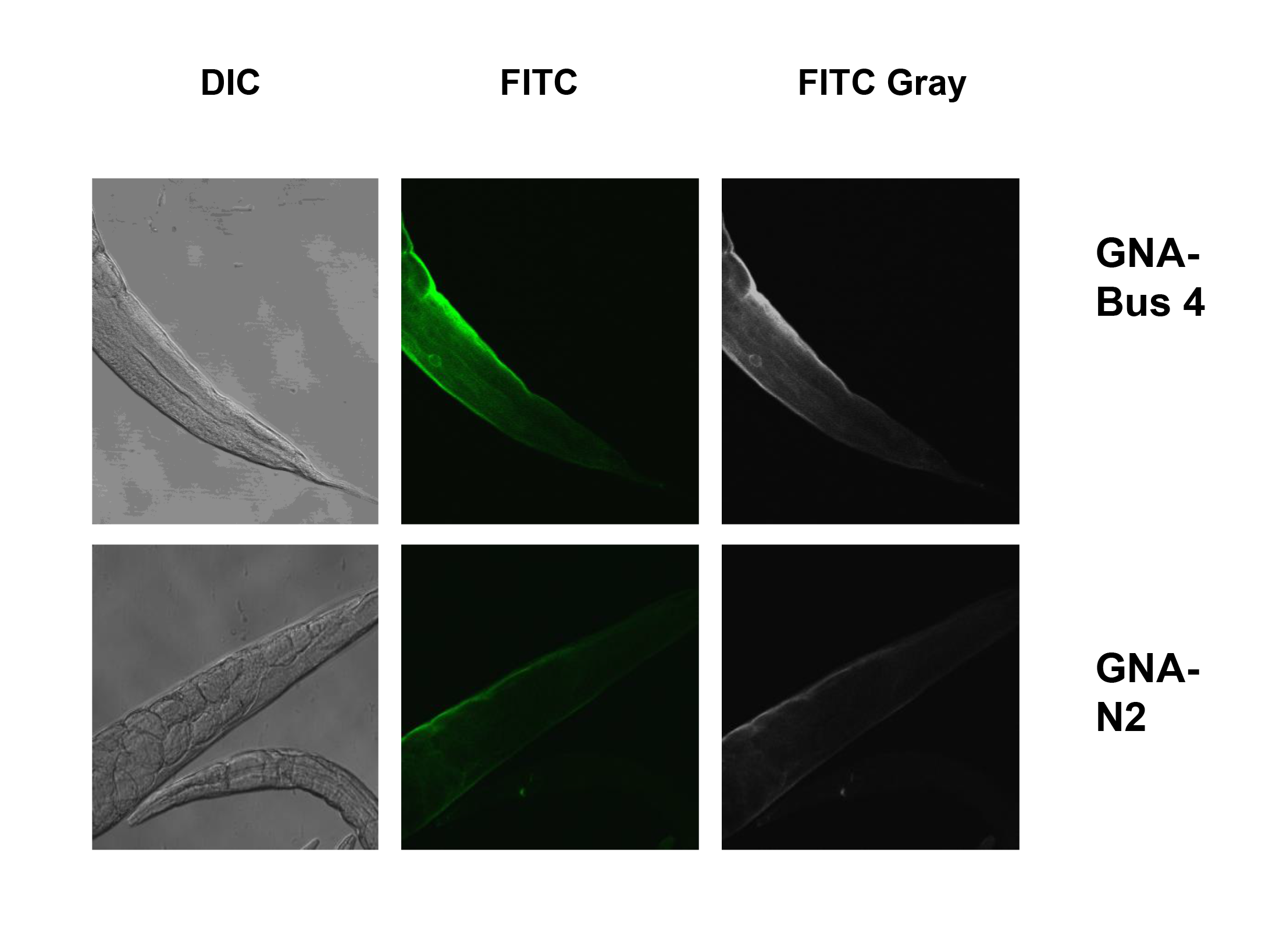

Supplement: Figure S17 — GNA staining of acetone fixed N2 and bus-4 nematodes. The images were collected using FITC conjugated GNA. Staining is more intense in the bus-4 nematodes and most pronounced in the tail. (TIF) [file pone.0107250.s017.tif]

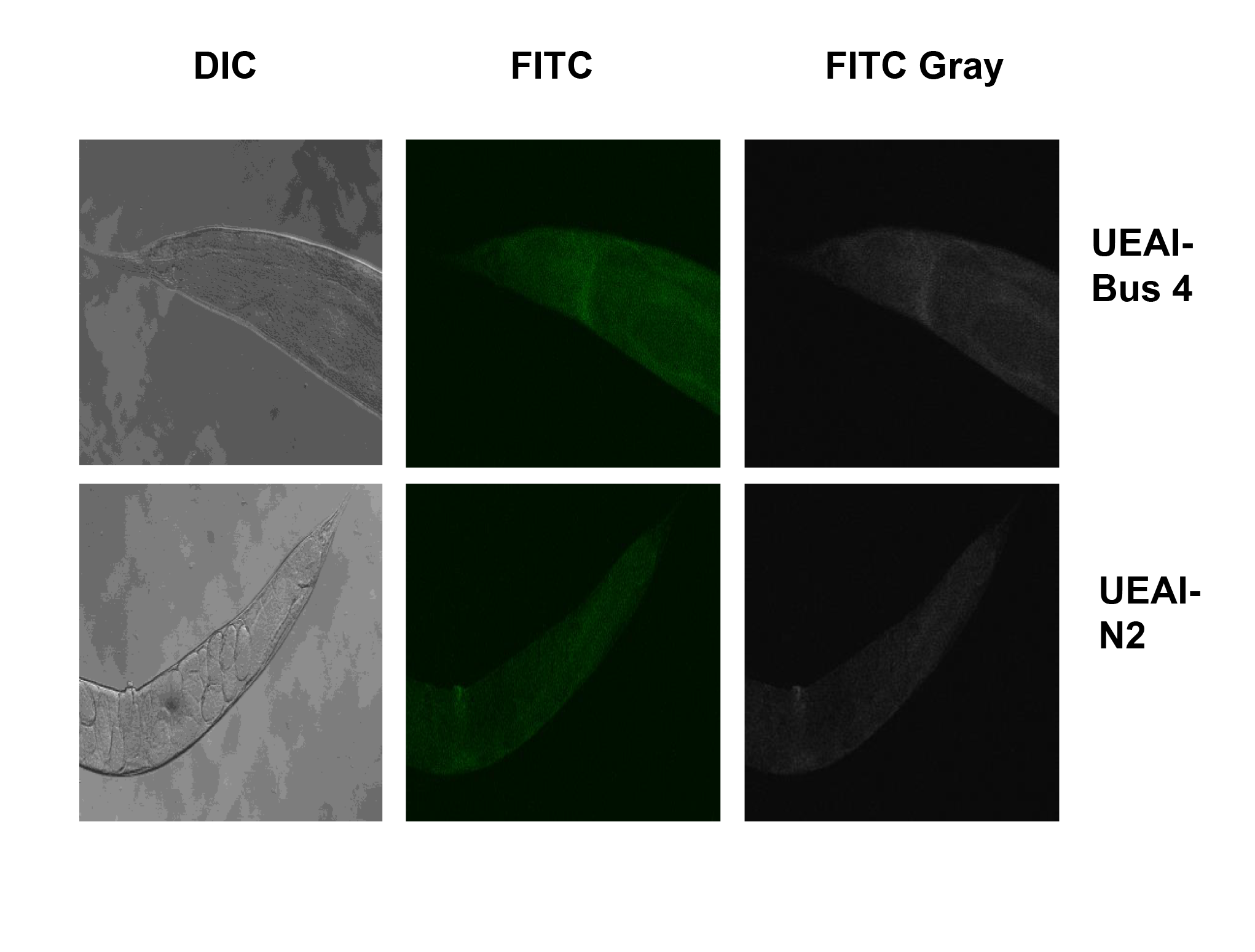

Supplement: Figure S18 — UEA-1 staining of acetone fixed N2 and bus-4 nematodes. The images were collected using FITC conjugated GNA. Staining is mildly more intense in the bus-4 nematodes near the tail region. (TIF) [file pone.0107250.s018.tif]

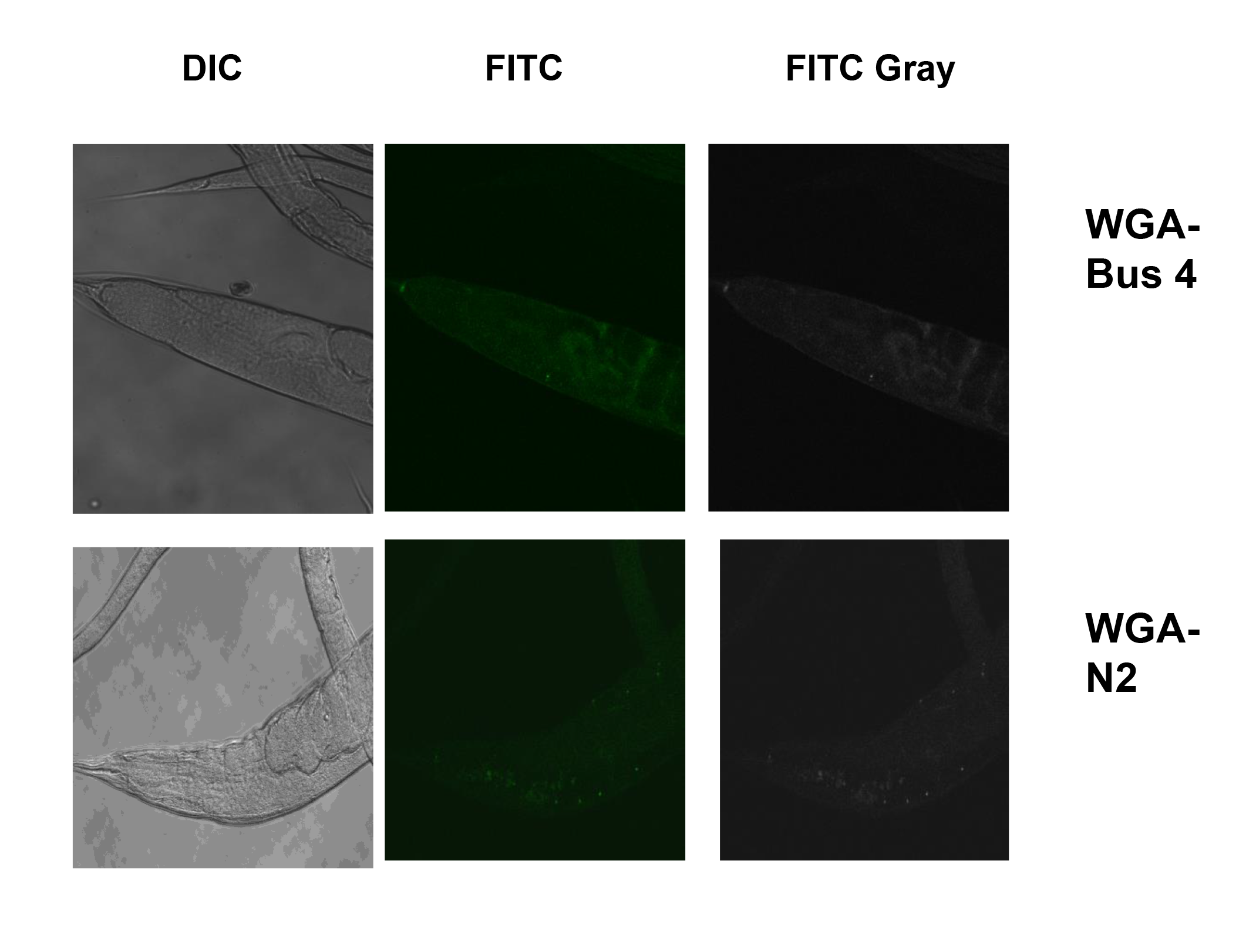

Supplement: Figure S19 — WGA staining of acetone fixed N2 and bus-4 nematodes. The images were collected using FITC conjugated WGA. Little to no difference in staining was observed. (TIF) [file pone.0107250.s019.tif]

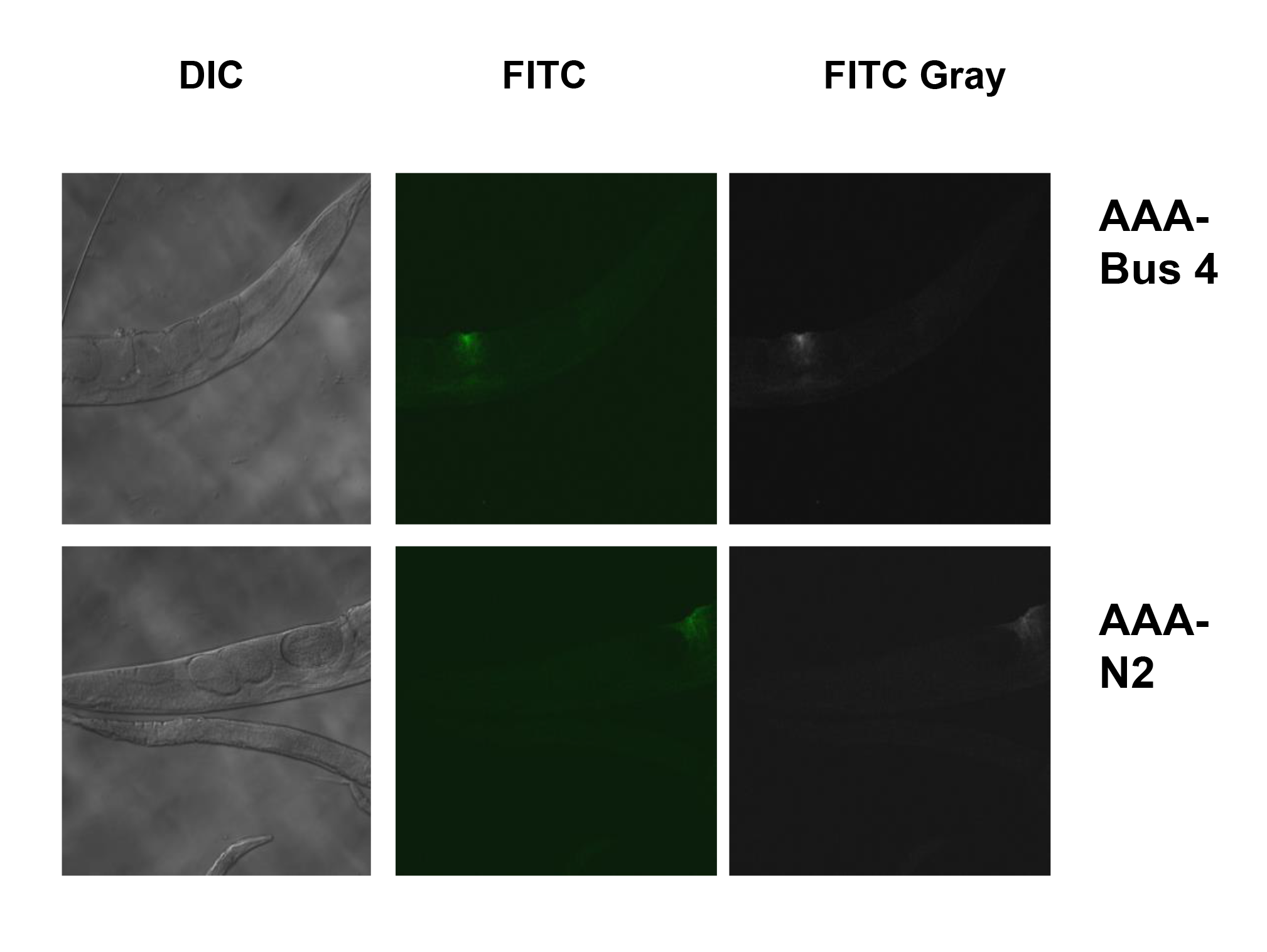

Supplement: Figure S20 — AAA staining of acetone fixed N2 and bus-4 nematodes. The images were collected using FITC conjugated AAA. Little to no difference in staining was observed. (TIF) [file pone.0107250.s020.tif]

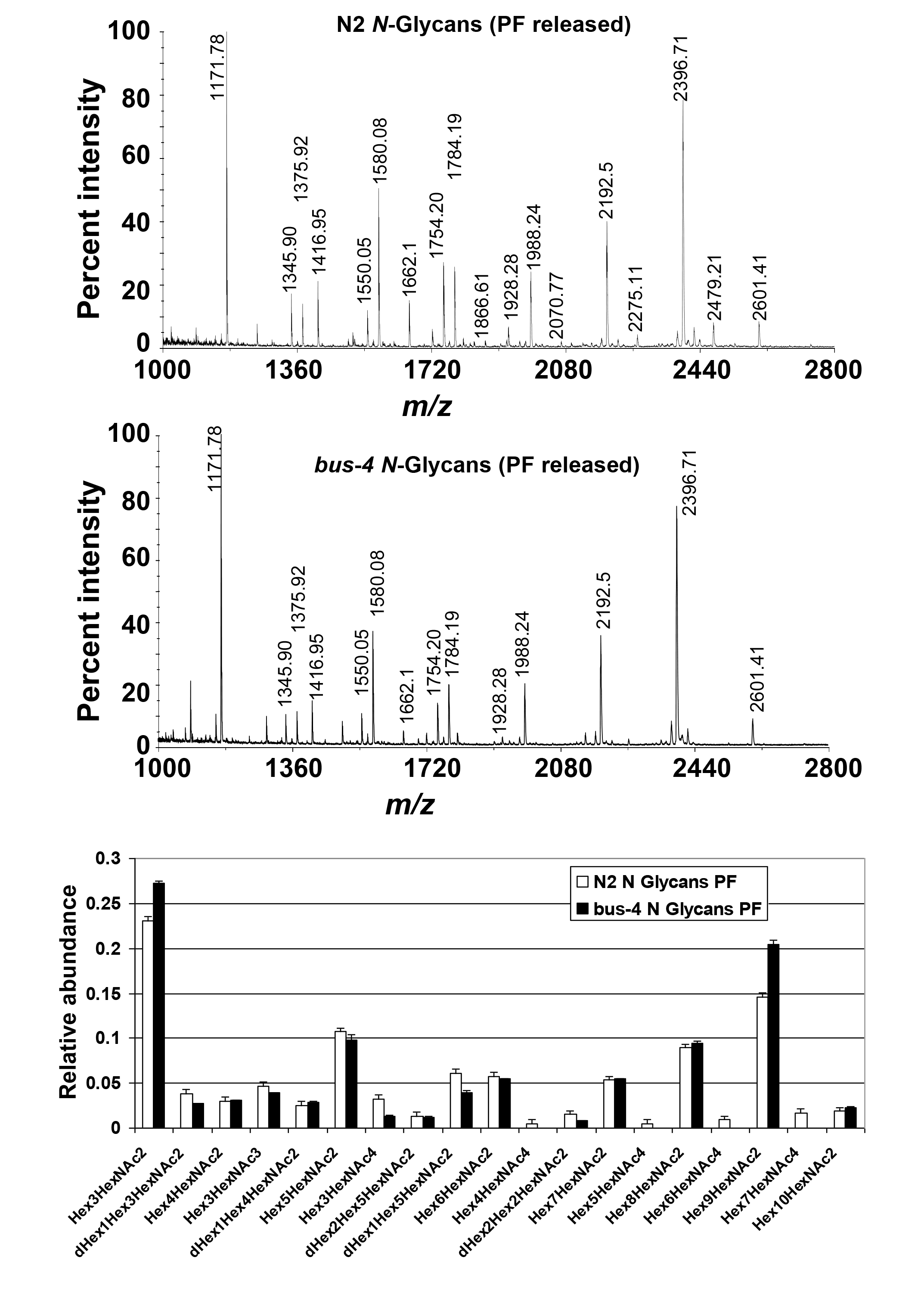

Supplement: Figure S21 — MALDI-TOF MS analysis of permethylated bus-4 and N2 PNGase F released N -glycans. Top panel: N2 PNGase F released glycans spectrum; Center panelbus-4 PNGase F released N-glycans; lower panel comparative histogram. (TIF) [file pone.0107250.s021.tif]

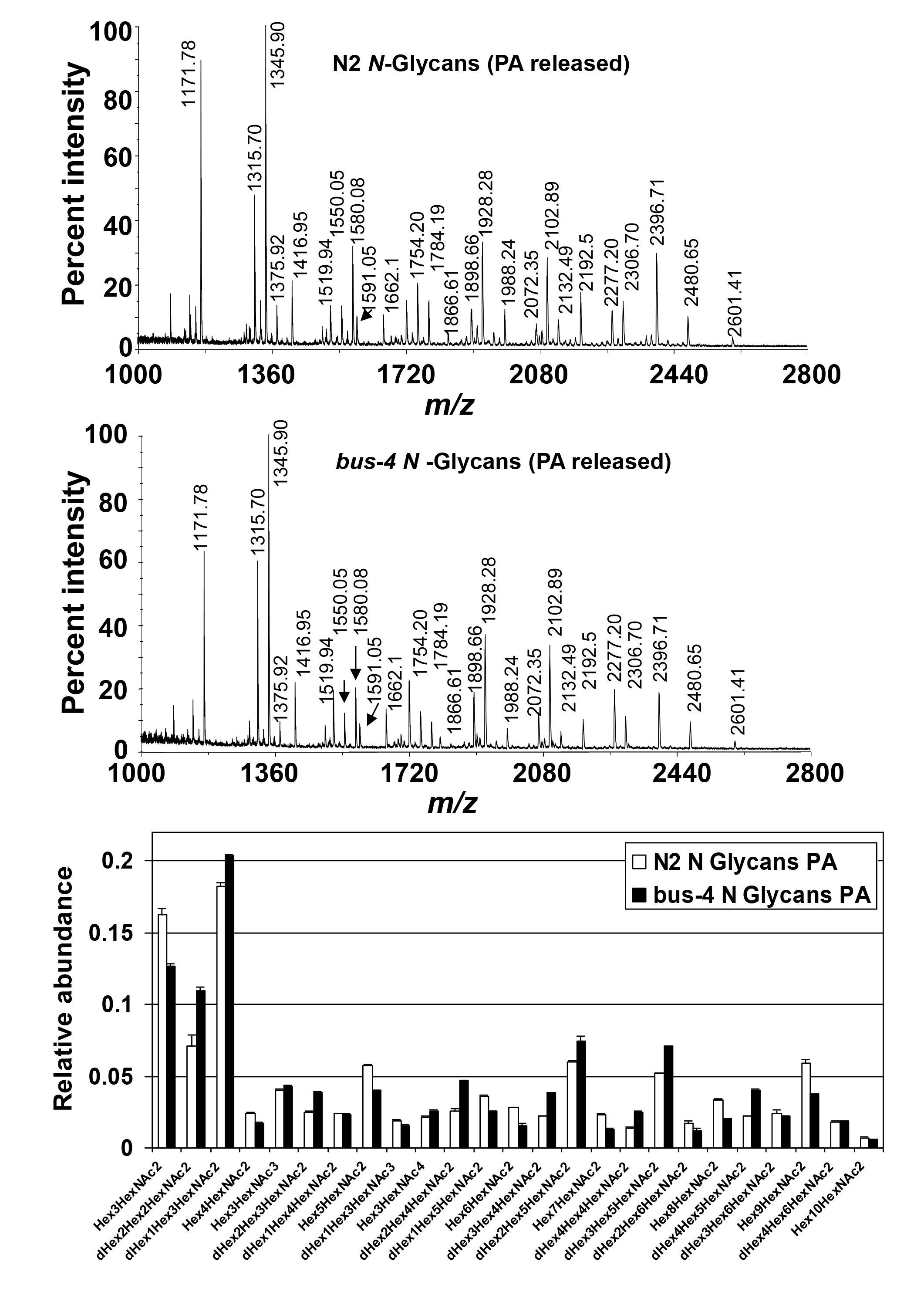

Supplement: Figure S22 — MALDI-TOF MS analysis of permethylated bus-4 and N2 PNGase A released N -glycans. Top panel: N2 PNGase F released glycans spectrum; Center panelbus-4 PNGase F released N-glycans; lower panel comparative histogram. (TIF) [file pone.0107250.s022.tif]
