# Supplementary material for: Caenorhabditis elegans Bacterial Pathogen Resistant bus-4 Mutants Produce Altered Mucins
Source: PLoS One. 2014 Oct 8;9(10):e107250. doi: 10.1371/journal.pone.0107250 (PMC4189790; doi:10.1371/journal.pone.0107250)
Supplement: Table S1 — aGlycans molecular ions that are only observed in PNGaseA releases. b All ions are sodium adducts. (DOC) [file pone.0107250.s023.doc]

Table S1: Composition of *C. elegans* permethylated *N*-glycans released from N2 wild type and the mutant strain *bus*-*4*. The data were obtained from the MALDI-TOF MS analysis, and all molecular ions are present in sodiated form ([M+Na]^+^).

| Measured *m/z* | Calculated *m/z* | Composition^b^ |
| --- | --- | --- |
| 1171.78  1315.70^a^  1345.90  1375.92  1416.95  1519.95  1550.05  1580.07  1591.05^a^  1662.10  1724.19  1754.20  1784.19  1866.28  1898.66^a^  1928.28  1988.25  2070.71  2072.35  2102.89^a^  2132.49^a^  2192.48  2274.05  2277.20^a^  2306.70^a^  2396.65  2479.18  2481.23^a^  2601.41 | 1171.58  1315.66  1345.67  1375.68  1416.71  1519.76  1549.77  1579.78  1590.80  1661.84  1723.86  1753.87  1783.88  1865.95  1897.95  1927.96  1987.98  2070.03  2072.04  2102.05  2132.06  2192.08  2274.13  2276.14  2306.15  2396.18  2478.23  2480.24  2600.28 | Hex_3_HexNAc_2_ dHex_2_Hex_2_HexNAc_2_ dHex_1_Hex_3_HexNAc_2_ Hex_4_HexNAc_2_  Hex_3_HexNAc_3_ dHex_2_Hex_3_HexNAc_2_ dHex_1_Hex_4_HexNAc_2_  Hex_5_HexNAc_2_ dHex_1_Hex_3_HexNAc_3_ Hex_3_HexNAc_4_ dHex_2_Hex_4_HexNAc_2_ dHex_1_Hex_5_HexNAc_2_ Hex_6_HexNAc_2_  Hex_4_HexNAc_4_ dHex_3_Hex_4_HexNAc_2_ dHex_2_Hex_5_HexNAc_2_ Hex_7_HexNAc_2_  Hex_5_HexNAc_4_ dHex_4_Hex_4_HexNAc_2_ dHex_3_Hex_5_HexNAc_2_ dHex_2_Hex_6_HexNAc_2_ Hex_8_HexNAc_2_  Hex_6_HexNAc_4_ dHex_4_Hex_5_HexNAc_2_ dHex_3_Hex_6_HexNAc_2_  Hex_9_HexNAc_2_  Hex_7_HexNAc_4_ dHex_4_Hex_6_HexNAc_2_ Hex_10_HexNAc_2_ |

^a^ Glycans molecular ions that are only observed in PNGaseA releases

^b^ All ions are sodium adducts
